# Genomic comparative analysis of *Ophiocordyceps unilateralis sensu* lato

**DOI:** 10.3389/fmicb.2024.1293077

**Published:** 2024-04-15

**Authors:** Yingling Lu, Dexiang Tang, Zuoheng Liu, Jing Zhao, Yue Chen, Jinmei Ma, Lijun Luo, Hong Yu

**Affiliations:** ^1^Yunnan Herbal Laboratory, College of Ecology and Environmental Sciences, Yunnan University, Kunming, China; ^2^The International Joint Research Center for Sustainable Utilization of Cordyceps Bioresources in China and Southeast Asia, Yunnan University, Kunming, China

**Keywords:** one new taxon, whole-genome sequence, secondary metabolite, biosynthesis gene cluster, gene mining

## Abstract

*Ophiocordyceps unilateralis sensu* lato is a common pathogenic fungus of ants. A new species, *O. fusiformispora*, was described based on morphology and phylogenetic evidence from five genes (SSU, LSU, *TEF1α*, *RPB1*, and *RPB2*). The whole genomes of *O. fusiformispora*, *O. contiispora*, *O. subtiliphialida*, *O. satoi*, *O. flabellata*, *O. acroasca*, and *O. camponoti-leonardi* were sequenced and annotated and compared with whole genome sequences of other species in *O. unilateralis sensu* lato. The basic genome-wide characteristics of the 12 species showed that the related species had similar GC content and genome size. AntiSMASH and local BLAST analyses revealed that the number and types of putative SM BGCs, NPPS, PKS, and hybrid PKS-NRPS domains for the 12 species differed significantly among different species in the same genus. The putative BGC of five compounds, namely, NG-391, lucilactaene, higginsianin B, pyripyropene A, and pyranonigrin E were excavated. NG-391 and lucilactaene were 7-desmethyl analogs of fusarin C. Furthermore, the 12 genomes had common domains, such as KS-AT-DH-MT-ER-KR-ACP and SAT-KS-AT-PT-ACP-ACP-Te. The ML and BI trees of SAT-KS-AT-PT-ACP-ACP-Te were highly consistent with the multigene phylogenetic tree in the 12 species. This study provided a method to obtain the living culture of *O. unilateralis sensu* lato species and its asexual formed on the basis of living culture, which was of great value for further study of *O. unilateralis sensu* lato species in the future, and also laid a foundation for further analysis of secondary metabolites of *O. unilateralis sensu* lato.

## 1 Introduction

**Figure 1:**
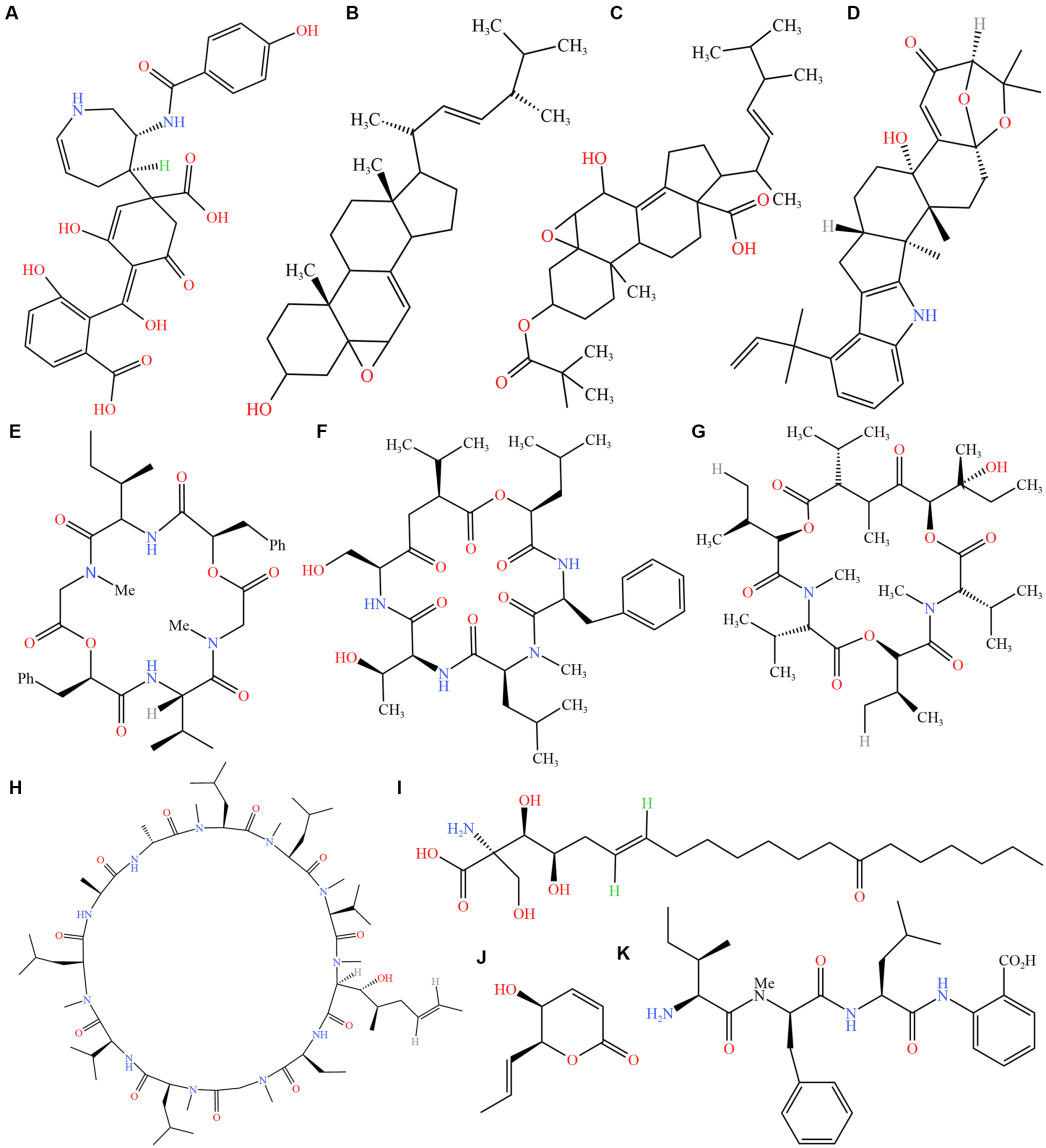
Structures of secondary metabolites from *Cordyceps sensu* lato. **(A)** ophiocordin; **(B)** erogosterol; **(C)** ergokonin **C**; **(D)** aflatrem; **(E)** hirsutellide **A**; **(F)** hirsutatins **A**; **(G)** enniatins **L**; **(H)** cyclosporin **A**; **(I)** myriocin; **(J)** phomalactone; **(K)** hirsutellic acid. Source: https://www.ncbi.nlm.nih.gov/pccompound, software: KingDraw (v3.0.2.20).

The classification system of *Cordyceps sensu* lato was widely accepted, and *Ophiocordyceps* was a genus in the family Ophiocordycipitaceae (Sung et al., 2007). The complex of *O. unilateralis* (Tul. and C. Tul.) Petch was the ubiquitous ant parasite fungus, “zombie ant fungus, or broadly *O. unilateralis* s. l.” (Evans et al., 2011a,b). The fungus could alter the behavior of ants. Controlling the ants to leave the nest to die, often in an exposed position where they are attached or bitten leaves or branches in a “death grip” (Hughes et al., 2011; Tang et al., 2023a). Bioactive compounds with neuroregulatory and physiological effects, as well as the destruction and excessive contraction of jaw muscle tissue, had been suggested as possible causes of host behavior disorder (Hughes et al., 2011; De Bekker et al., 2014; Fredericksen et al., 2017; Kobmoo et al., 2018; Loreto and Hughes, 2019; Mangold et al., 2019). *Cordyceps sensu* lato had many bioactive components, such as hirsutellic acid A (Thongtan et al., 2006), enterotoxins (De Bekker et al., 2017), ophiocordin (Kneifel et al., 1977), myriocin (Cheng et al., 2017), enniatins L (Hu and Li, 2007), cyclosporin A (Agathos and Parekh, 1990), hirsutatins A (Isaka et al., 2005), aflatrem (Will et al., 2020), leucinostatins (Bernatchez et al., 2022), phomalactone (Prathumpai and Kocharin, 2016), ergokonin C (Gräfe et al., 1991), erogosterol (Shin et al., 2021) and hirsutellide A (Vongvanich et al., 2002; Figure 1). These compounds worked in a variety of ways. For example, Will et al. (2020) proposed that differentially expressed genes tied to ant neurobiology, odor response, circadian rhythms, and foraging behavior may result by the activity of putative fungal effectors such as enterotoxins, aflatrem, and mechanisms, disrupting feeding behaviors in ant. Cordycepin treated mice that are infected with *Trypanosoma evansi* (Dalla Rosa et al., 2013). Myriocin treatment significantly attenuated liver pathology including steatosis, lobular inflammation, and ballooning (Yang et al., 2019). Oosporein, a red 1,4-bibebzoquinone derivative, exhibited antifungal, antiviral, antibiotic, and insecticidal activities (Feng et al., 2015).

Natural products, also known as secondary metabolites (SMs), had played an important role in the history of drug discovery and its development (Chu et al., 2020). As more and more microbial genomes are sequenced, bioinformatics analysis had revealed a vast resource of novel SMs. Genome mining was a new strategy of SM discovery based on gene cluster sequences and biosynthetic pathways. At the same time, it could directly associate the structures of SMs with synthetic pathways and facilitate the study of biosynthesis and combinatorial biosynthesis (Li et al., 2018). Some analysis tools and procedures (e.g., antibiotics and Secondary Metabolites Analysis Shell, antiSMASH; ClusterFinder; Antibiotic Resistant Target Seeker, and ARTS) allowed for rapid and direct detection of biosynthetic gene clusters (BGCs), as well as the diversity of gene cluster families, such as polyketide synthases (PKSs), non-ribosomal peptide synthetases (NRPSs), and terpene synthases (TSs). The zombie ant fungus SMs had been found to engage in manipulative behavior. *O. camponoti-floridani* contained homologous genes that had been identified as producing SMs in *O. kimflemingiae* (De Bekker et al., 2015; Will et al., 2023 print). Previously, De Bekker et al. (2015) identified one possible product of this cluster to be an ergot alkaloid based on a tryptophan dimethylallyltransferase backbone gene of the cluster. Nicholson et al. (2009) characterized aflatrem biosynthesis gene loci and BGC. Will et al. (2020) found that cluster 18 in the genomes of *O. camponoti-floridani* and *O. kimflemingiae* was highly consistent with the afltrem gene cluster. The fusarin C gene cluster consisted of nine genes *(fus1-fus9)* that were co-expressed under high nitrogen and acidic pH conditions. The synthesis of fusarin C appeared to require four key genes, namely, *fus1, fus2, fus8, and fus9* (Niehaus et al., 2013). Fusarin C might act as a virulence factor to disrupt ant physiology and behavior. The genomes of *O. kimflemingiae* and *O. polyrhachis-furcata* both contained homologs of genes necessary for the synthesis of fusarin C (De Bekker et al., 2015; Wichadakul et al., 2015). Aflatoxin and fusarin C, SMs of fungi, were involved in host ant manipulation, and evidence of gene clusters that might produce their analogs was found in the two zombie ant fungi by genome mining.

However, whether other zombie ant fungi also had the potential to produce aflatoxin, fusarin C, or similar SM BGCs or had the potential to produce other SM BGCs not reported in this group. In this study, the species *O. fusiformispora* Hong Yu bis & D.X. Tang, Y.L. Lu, sp. nov. was first introduced. To discover more potential gene clusters of SMs that might be related to ant manipulative behavior, whole genomes of *O. contiispora* Hong Yu bis & D.X. Tang, *O. subtiliphialida* Hong Yu bis & D.X. Tang, *O. satoi* Araújo, H.C. Evans & D.P. Hughes, *O. flabellata* Hong Yu bis & D.X. Tang, *O. acroasca* Hong Yu bis & D.X. Tang, *O. camponoti-leonardi* Kobmoo, Mongkols., Tasan., Thanakitp. & Luangsa-ard, *O. fusiformispora* were sequenced and annotated, as well as basic characteristics and gene mining were compared with other species of *O. unilateralis* complex (i.e., *O. camponoti-rufipedis* H.C. Evans & D.P. Hughes, *O. camponoti-floridani* Araújo, H.C. Evans & D.P. Hughe*s*, *O. polyrhachis-furcata* Kobmoo, Mongkols., Tasan., Thanakitp. & Luangsa-ard, *O. camponoti-saundersi* Kobmoo, Mongkols., Tasan., Thanakitp. & Luangsa-ard and *O. unilateralis* (Tul. and C. Tul.) Petch; Supplementary Figure S1). This will provide additional insights into the co-evolutionary relationships and precise manipulation mechanisms of the *O. unilateralis* complex and host ants and into the neurobiology of novel bioactive compounds and animal behavior.

## 2 Materials and methods

### 2.1 Test materials

*O. contiispora*, *O. subtiliphialida*, *O. satoi*, *O. flabellata*, *O. acroasca*, and *O. camponoti-leonardi* were the known species in our group (Tang et al., 2023a,b). *O. fusiformispora* strain was collected from Puer City, Yunnan Province, China. The voucher specimens were stored in Yunnan Herbal Herbarium (YHH) of Yunnan University, and the isolated strains were stored in Yunnan Fungal Culture Collection (YFCC) of Yunnan University. The genome-wide data of *O. camponoti-rufipedis*, *O. camponoti-floridani*, *O. polyrhachis-furcata*, *O. camponoti-saunders*, and *O. unilateralis* were obtained from NCBI,^1^ accession numbers GCA_002591395.1, GCA_012980515.1, GCA_001633055.2, GCA_003339415.1, and GCA_001272575.2, respectively.

### 2.2 Morphological observations

The sexual morph observation was performed by photographing and measuring the ascomata using Olympus SZ61 stereomicroscope (Olympus Corporation, Tokyo, Japan). Freehand or frozen sections of the structure of the fruit body were placed in lactophenol cotton blue solution for microscopic study and photomicrography. The frozen sections were used by freezing Microtome HM525NX (Thermo Fisher Scientific, Massachusetts, United States). The micro-morphological characteristics of fungi (perithecia, asci, apical caps, and ascospores) were examined using Olympus CX40 and BX53 microscopes. Two methods were used to observe asexual morphology. One was observed directly from stromata, sutures, legs, and joints of the specimen, and the other was observed in pure culture on PPDA solid medium (20 g/L Potato Powder, 10 g/L Yeast powder, 20 g/L Glucose, and 18 g/L Agar Powder, 1 L H_2_O). Culture on solid medium plates was incubated at 25°C for 30–40 days and photographed with a Canon 750 D camera (Canon Inc., Tokoy, Japan). The solid medium was made 0.5–1 mm thick and then spited into 5 mm long and 5 mm wide sections. Finally, the medium was placed on a glass slide in a sterile culture dish (with a glass rod buffer so that it could not be submerged in sterile water). The colony was placed on a solid medium, lightly covered with a cover slide, added with 3 mL of sterile water, and left at 25°C for 30–40 days. The BX53 microscope and Olympus CX40 were used to study the asexual characteristics of conidiogenous cells, conidiophores, and conidia.

### 2.3 DNA extraction, polymerase chain reaction, and sequencing

Specimens and axenic living cultures were prepared for DNA extraction. Total DNA was extracted using the CTAB method as described by Yi et al. (2001). Five genes (SSU, LSU, TEF, *RPB1*, and *RPB2*) were amplified and sequenced. The fraction of nuclear ribosomal small subunit (SSU) was amplified by primer pair NS1 and NS4 (White et al., 1990). The nuclear ribosomal large subunit (LSU) was amplified by primer pair LROP (Hopple, 1994) and LR5 (Vilgalys and Hester, 1990). The primer pair 2218R and 983F was used to amplify the translation elongation factor 1α (TEF) (Rehner and Buckley, 2005). The largest and second largest subunits of RNA polymerase II (*RPB1* and *RPB2*) were amplified by primer pairs RPB1 and RPB1Cr_oph and fRPB2-7cR and fRPB2-5F, respectively (Liu et al., 1999; Castlebury et al., 2004; Araújo et al., 2018). The polymerase chain reaction (PCR) matrix was carried out in 25 μL final volume, consisting of 17.25 of μl sterile water, 2 μL of dNTP (2.5 mmoL/L), 2.5 μL of PCR 10 × Buffer (2 mmoL/L Mg^2+^) (Transgen Biotech, Beijing, China), 1 μL of forwarding primers (10 μmol/L), 0.25 μL of Taq DNA polymerase (Transgen Biotech, Beijing, China), 1 μL of reverse primers (10 1 μmol/L), and 1 μL of DNA template (500 ng/μL). The amplification reactions were performed in BIO-RAD T100TM thermal cycler (BIO-RAD Laboratories, Hercules, CA, United States). The PCR procedure for five genes is described by Wang et al. (2020). The Beijing Genomics Institute (Chongqing, China) performed the target gene amplification and sequencing.

### 2.4 Genome sequencing and assembly

*Ophiocordyceps contiispora*, *O. subtiliphialida*, *O. satoi*, *O. flabellata*, *O. acroasca*, *O. camponoti-leonardi*, and *O. fusiformispora* strains were cultured on PPDA solid medium (20 g/L Potato Powder, 10 g/L Yeast powder, 20 g/L Glucose, 18 g/L Agar Powder, and 1 L H_2_O) at 25°C for 60 days. The mycelium was transferred to PPA liquid medium (20 g/L Potato Powder, 10 g/L Yeast powder, 20 g/L Glucose, and 1 L H_2_O) at 25°C static cultivation for 2–3 months. Appropriate amounts of *O. contiispora*, *O. subtiliphialida*, *O. satoi*, *O. flabellata*, *O. acroasca*, *O. camponoti-leonardi*, and *O. fusiformispora* mycelium were scraped, respectively, and the total genomic DNA was extracted using ZR Fungal DNA Kit (Catalog number D6005) and then sequenced on an automatic sequence analyzer (BGI Co., Ltd., Wuhan, China) using the same primers as those used in amplification. Illumina NovaSeq 6,000 (Nanopore, Wuhan, China) high-throughput sequencing platform was adopted to construct gene library with 150-bp insertion fragment, respectively. Raw image data files obtained from high-throughput sequencing were converted to raw reads by Base Calling analysis. The software fastp (v0.21.0) was used to filter the raw reads, discard low-quality reads, and obtain clean data. To obtain high-quality collication-free genomic assembly readings, AdapterRemoval (v2) and SOAPec (v2.0) were used to filter the raw data. A5-MiSeq and SPAdes were used to construct contig and scaffold. Finally, the assembly effects of contig and scaffold were evaluated using pilon v1.18 software.

### 2.5 Gene prediction and annotation

A combination of homologous, *de novo*, and transcript was used for gene prediction. Based on the existing database on gene function and metabolic pathways, the predicted genes were annotated by BLAST search, including Kyoto Encyclopedia of Genes and Genomes (KEGG), NCBI non-redundant protein sequences (NR), Gene Ontology (GO), Cluster of Orthologous Groups of eukaryotic complete genomes (KOG), Pfam, and Interpro.

### 2.6 Analysis of secondary metabolite biosynthesis gene cluster

The antiSMASH^2^ online program was used to perform gene cluster prediction at the level of 7 genomic scaffolds of *O. unilateralis* complex. Based on antiSMASH-detected scaffolds with gene clusters, the online program FGENESH^3^ was used to predict gene structures using *O. sinensis* as a parameter. To obtain the domain, the PKS/NRPS online program^4^ was used to predict gene clusters in contigs where NRPS/PKS genes were located. At the same time, the online program Protein BLAST^5^ was used for NRPS/PKS genes of contig protein ratio analysis.

### 2.7 Cluster analysis

The known PKS, hybrid PKS-NRPS protein sequences, and polygene nucleotide sequences (SSU, LSU, TEF, *RPB1*, and *RPB2*) were downloaded from NCBI and compared with these sequences using the Clustal W program in the MEGA5.0 software for multi-sequence comparison. Based on five-gene dataset, software PhyloSuite (v1.2.2 Win) was used to construct phylogenetic tree of the Maximum Likelihood (ML) and the Bayesian Inference (BI), and software PhyloSuite (v1.2.2 Win) was used to construct clustering tree of the NR-PKS gene dataset. In addition, using the method from the online program IQ - TREE web server,^6^ since the report 1,000, the rest of the default parameter was used to construct the ML cluster analysis tree of PKS or hybrid PKS-NRPS proteins between *O. unilateralis* complex species and other fungi.

## 3 Results and analysis

### 3.1 Identification of strain *Ophiocordyceps fusiformispora* Hong Yu bis and D. X. Tang, Y. L. Lu, sp. nov.

#### 3.1.1 Phylogenetic analyses

Based on the joint matrix of nucleotide sequences of nrSSU, nrLSU, TEF, *RPB1*, and *RPB2*, the molecular phylogenetic tree of *Ophiocordyceps* was reconstructed by the ML and BI. The phylogenetic tree, represented by *Tolypocladium inflatum* OSC 71235 and *Tolypocladium ophioglossoides* CBS 100239 as the out group, consisted of four clades of the genus *Ophiocordyceps*: *Hirsutella* clade, *O. sphecocephala* clade, *O. sobolifera* clade, and *O. ravenelii* clade, with 129 sequences, of which 30 single gene fragments were self-detected (Supplementary Table S1). Among the 129 sequences, *O. albacongiuae*, *O. aphodii*, *O. australis*, *O. basiasca*, *O. brunneipunctata*, *O. buquetii*, *O. camponoti-chartificis*, *O. camponoti-femorati*, *O. camponoti-hippocrepidis*, *O. camponoti-indiani*, *O. camponoti-nidulantis*, *O. citrina*, *O. clavata*, *O. cochlidiicola*, *O. curculionum*, *O. daceti*, *O. formicarum*, *O. formosana*, *O. ghanensis*, *O. kniphofioides*, *O. lloydii*, *O. myrmecophila*, *O. naomipierceae*, *O. neovolkiana*, *O. nigrella*, *O. nutans*, *O. oecophyllae*, *O. ponerinarum*, *O. pulvinata*, *O. purpureostromata*, *O. ravenelii*, *O. sinensis*, *O. sobolifera*, *O. sphecocephala*, *O. tricentri*, *O. yakusimensis*, *T. inflatum*, and *T. ophioglossoides* were used with one strain sequence each. *O. acicularis*, *O. blakebarnesii*, *O. camponoti-balzani*, *O. camponoti-bispinosi*, *O. camponoti-floridani*, *O. camponoti-novogranadensis*, *O. camponoti-renggeri*, *O. camponoti-rufipedis*, *O. camponoti-saundersi*, *O. dipterigena*, *O. flabellata*, *O. forquignonii*, *O. halabalaensis*, *O. kimflemingiae*, *O. konnoana*, *O. lilacina*, *O. longissima*, *O. melolonthae*, *O. monacidis*, *O. nooreniae*, *O. nuozhaduensis*, *O. odonatae*, *O. ootakii*, *O. polyrhachis-furcata*, *O. rami*, *O. rhizoidea*, *O. septa*, *O. stylophora*, *O. tianshanensis*, and *O. unilaterali* used two strain sequences each. Three strains of *O. acroascas*, *O. camponoti-leonardi*, *O. irangiensis*, and *O. satoi* were used, respectively. *O. contiispora* and *O. subtiliphialida* used four strain sequences, *O. fusiformispora* used five strain sequences, and *O. bifertilis* used six strain sequences.

**Figure 2:**
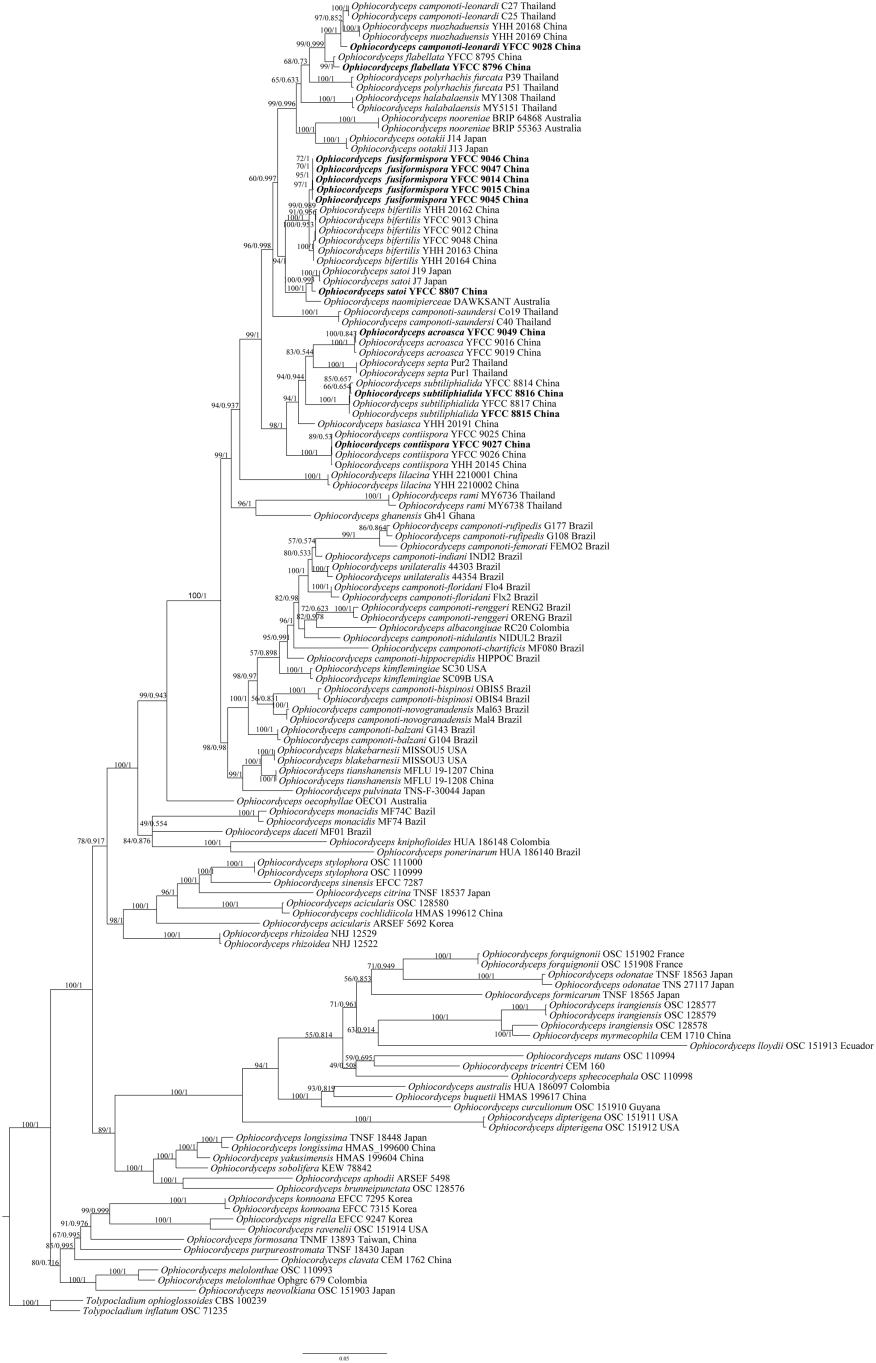
The phylogenetic tree of *Ophiocordyceps* and its related genera was inferred from five-gene dataset (SSU, LSU, TEF, *RPB1*, *RPB2*) based on the Bayesian inference and the Maximum likelihood analyses. Each value at a node indicates a bootstrap proportion (the left) and Bayesian posterior probability (the right). *Tolypocladium inflatum* OSC 71235 and *Tolypocladium ophioglossoides* CBS 100239 were designated as outgroups.

The matrix had 2,601 distinct patterns, 1,630 parsimony-informative, 403 singleton sites, and 2,901 constant sites. The best-fit model obtained from the 88 DNA models in ModelFinder was TIM2 + F + I + G4 for the ML analysis. The IQ-tree of the best score found was −50698.515, and the total tree length was 4.114. The best-fit model obtained from the 88 DNA models in ModelFinder was GTR + F + I + G4 for Mrbayes analysis. The IQ-tree of the best score found was −50698.515, and the total tree length was 4.114, The tree shape obtained by the ML method was basically consistent with the BI. The paper showed that the tree shape was a phylogenetic tree by the maximum likelihood method (Figure 2).

From the perspective of a five-gene phylogenetic tree, the four branches of *Ophiocordyceps* received high support rates. The cluster of *O. fusiformispora* collected and described in this study was in the core clade of *O. unilateralis* complex. It was clustered into a large clade with *O. unilateralis* complex species reported from Asian (Japan, Thailand), African (Ghana) and Oceania (Australia) countries, indicating a close genetic relationship. This was consistent with the results of Tang et al. (2023b). *O. fusiformispora* and *O. bifertilis* were sister species to each other and formed a separate clade. Bootstrap promotions (BP) and Bayesian posterior probabilities (BPP) were 100%, and the topological structure was stable. Meanwhile, *O. fusiformispora*, *O. bifertilis*, and *O. satoi* were genetically closely related and the host were all *Polyrhachis* sp.

#### 3.1.2 Taxonomy

MycoBank: MB 850756.

Etymology: fusiformi- = fusiform, spora = spore, the epithet refered to fusiform conidia.

Holotype: YHH 20157.

**Figure 3:**
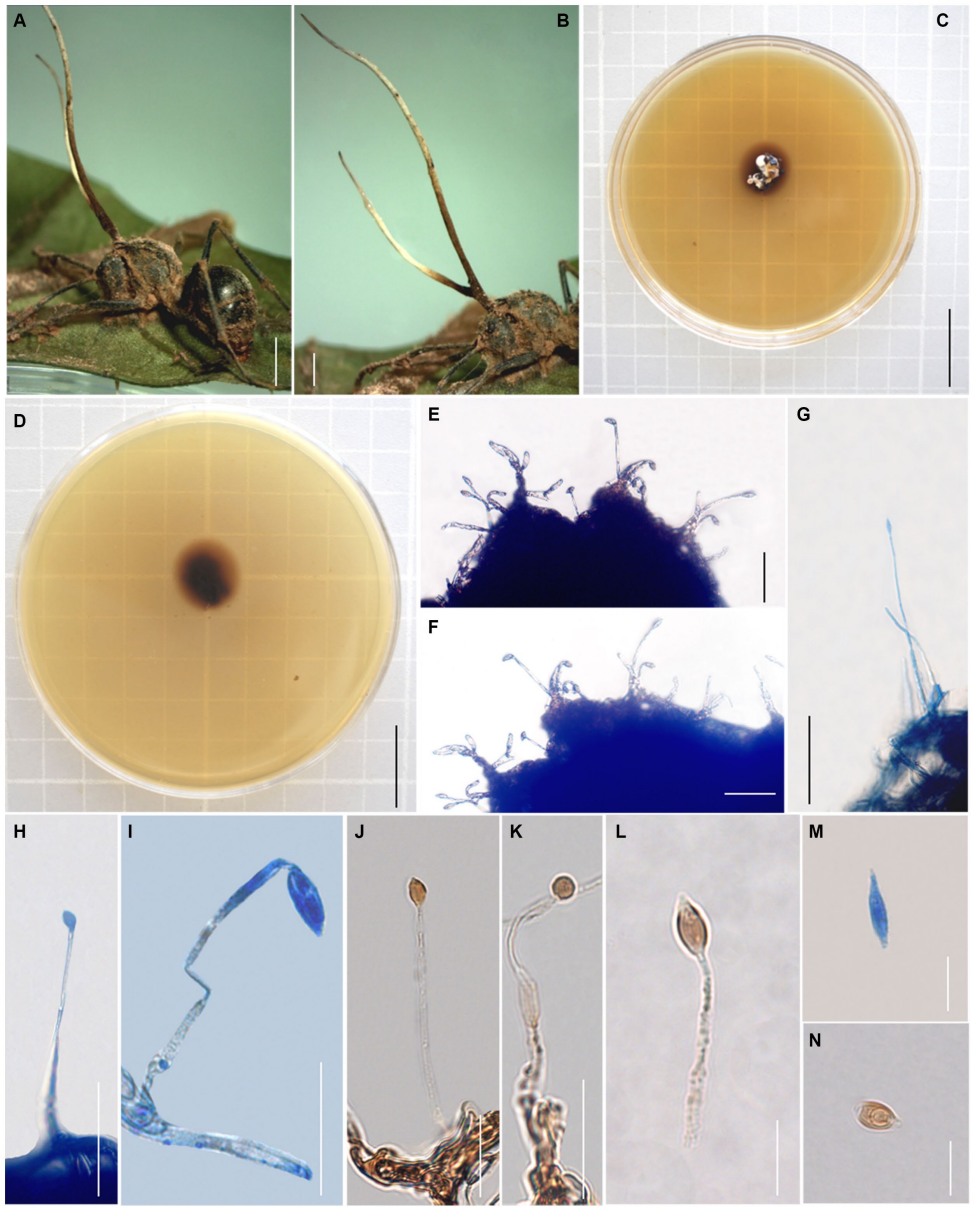
Morphology of *Ophiocordyceps fusiformispora*. **(A,B)** Polyrhachis ants was bited into a leaf of sapling; **(C,D)** Colonies on PDA medium; **(E–L)** Phialides andconidia; **(M,N)** conidia. Scale bars: **(A)** = 3,000 μm; **(B)** = 3,000 μm; **(C)** = 2 cm; **(D)** = 2 cm; **(E–H)** = 50 μm; **(I–L)** = 20 μm; **(M,N)** = 10 μm.

Sexual morph: External mycelia rarely produced from legs and sutures of the host. Stromata single, produced from dorsal pronotum of the ant, clavate, light brown, part bifurcate. Sexual morph were not observed (Figure 3).

Asexual morph: Colonies on PDA medium slow-growing, 8–18 mm diameter in 21 days at 25°C, milky white to light brown, raising cottony-shaped mycelia, reverse light brown to dark brown. Hyphae immersed in the medium, branched, septate, smooth-walled, hyaline. *Hirsutella* type-A observed from stromata and living culture. Phialides cylindrical, forming on hyphae, smooth, slightly swollen base, (22–) 26–79 × 2.3–5.1 μm, tapering abruptly a long neck, 0.6–2 μm wide. Conidia fusiform, hyaline, smooth-walled, 4–9 × 2–5 μm.

Germination process: No germination was observed due to the specimens being immature.

Host: *Polyrhachis* sp. (Formicinae).

Type locality: China (Yunnan Province, Puer City).

Habitat: Infected *Polyrhachis* sp. was collected from evergreen broad-leaf forest, and was bited into a leaf of the sapling, from 0.5 to 1 m above the ground.

Distribution: China, Yunnan Province.

Material examined: China: Yunnan, Yunnan Province, Puer City, Sun River National Park. Infected *Polyrhachis* sp. was collected from evergreen broad-leaf forest, and was bited into a leaf of the sapling, from 0.5 to 1 m above the ground, at alt. 1487 m, 2°20′24″ N, 101°6′43″ E, 18 August 2020, Hong Yu bis (holotype: YHH 20157; ex-holotype living culture: YFCC 9015; YFCC 9045, YFCC 9046 and YFCC 9047).

Notes: *Ophiocordyceps fusiformispora* was characterized by infected *Polyrhachis* ant biting onto a leaf of the sapling, producing clavate stromata from dorsal pronotum of the ant, milky white to light brown colonies, *Hirsutella* type-A, cylindrical phialides, and forming fusiform conidia. Phylogenetic analyses showed that *O. fusiformispora* formed a sister lineage with *O. bifertilis*, was clustered in the *O. unilateralis* complex, with statistical supported from bootstrap proportions (BP = 100%) and Bayesian posterior probabilities (BPP = 100%). *Ophiocordyceps fusiformispora* was similar to *O. bifertilis* in the same host *Polyrhachis*, and *Hirsutella* type-A. However, it differed from *O. bifertilis* in that it produced cylindrical phialides, slightly swollen base, and fusiform conidia.

### 3.2 Basic genomic characteristics of seven species in *Ophiocordyceps unilateralis* complex

#### 3.2.1 Genome sequencing and assembly

The Illumina sequencing produced 21,859,916 raw reads and 21,795,740 high-quality reads of *O. fusiformispora* (Supplementary Table S2). The genome size of *O. fusiformispora* was 51.00 Mb, containing 41.81% GC content, 6,706 protein-coding genes, along with 90 tRNA, 15 rRNA and 20 snRNA. It showed that the quality of genome assembly was good. Among the 7 species in the *O. unilateralis* complex, the genomes showed significant differences in genome size (27.80–51.00 Mb) and GC content (41.81–53.22%), but the difference in total gene number (6,700–6,974) was small. Among *O. satoi* had the largest genome size (75.05 Mb), while *O. fusiformispora* had the smallest GC content (41.81%). *O. subtiliphialida*, had the smallest genome size (27.80 Mb) and the largest GC content (53.22%). This suggested significant differences at the genome level even among related species. These results also indicated that the more closely related the species, the smaller the differences in genome size, GC content, and total gene number. Meanwhile, *O. satoi* and *O. fusiformispora* were clustered together in phylogenetic tree, and the host of both species was *Polyrhachis* sp. *O. satoi* and *O. fusiformispora* had large genome size (75.05–51.00 Mb). It had been shown that genome sizes and GC contents was closely related to phylogeny.

#### 3.2.2 Genome annotation

**Figure 4:**
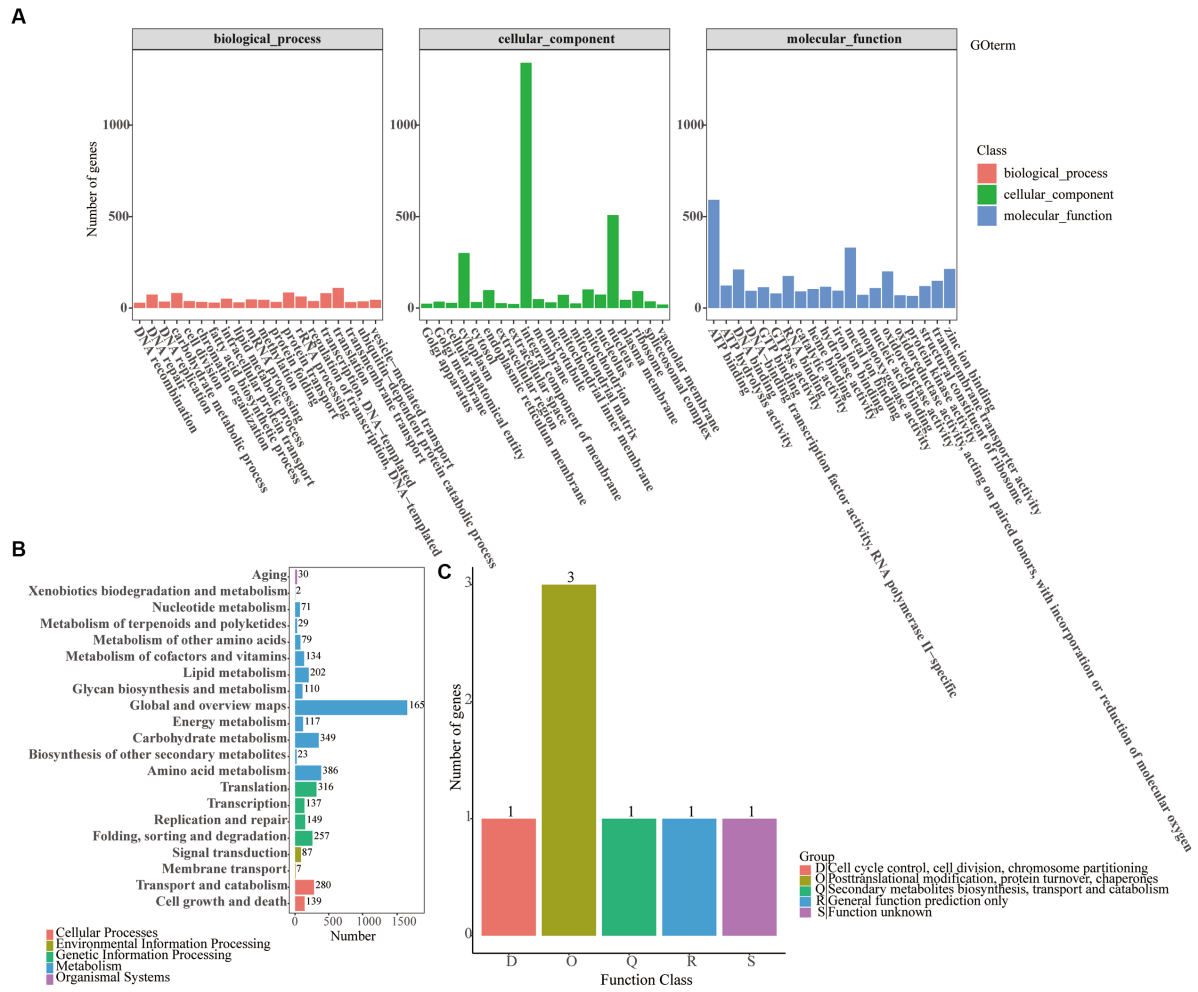
Functional annotation of *O. fusiformispora* genes encoding the proteins. **(A)** GO, **(B)** KEGG, **(C)** KOG.

Similarly, an analysis of 6,706 non-redundant *O. fusiformispora* genes in a publicly available protein sequence database produced mixed results, including the KEGG databases (2,131 genes/31.78%), the NR databases (6,690 genes/99.76%), the GO database (5,112 genes/76.23%), the KOG databases (5 genes/0.07), the Pfam databases (5,099 genes/76.04%) and the Interpro databases (6,546 genes/97.61) (Supplementary Table S3). And the other six species in *O. unilateralis sensu* lato, the KEGG (2,104–2,153 genes), NR (6,687–6,946 genes), GO (5,041–5,258 genes), KOG (3–10 genes), Pfam (5122–5,257 genes) and Interpro (6,540–6,800 genes) databases contained little difference in the number of genes each had. GO annotations of the genomes of 7 species of the *O. unilateralis* complex all indicated that translation, protein transport and carbohydrate metabolism processes were abundant genes in biological process (Figure 4A; Supplementary Figures S2–S7). The GO annotation revealed integral component of membrane, nucleus and cytoplasm from the cellular component, and ATP binding, metal ion binding and zinc ion binding from molecular function. The 7 species of *O. unilateralis sensu* lato were wild strain, in which many metabolic genes might be involved in signal transduction.

A comparison of the KEGG databases in 7 species of *O. unilateralis* complex showed that most of the predicted genes all were functionally associated with global and overview maps, amino acid metabolism and carbohydrate metabolism, and the predicted genes ranged from 1,629–1,668, 367–386, and 339–355, respectively (Figure 4B; Supplementary Figure S8). These findings suggested the presence of a rich diversity of lipid and protein metabolic functions that might make secondary metabolism more efficient. Furthermore, according to the KOG database, the functional genes predicted by *O. unilateralis sensu* lato 7 genomes varied enormously. *O. acroasca*, *O. contiispora*, *O. subtiliphialida*, *O. satoi*, *O. flabellata*, *O. fusiformispora*, *O. acroasca* and *O. camponoti-leonard* captured KOG genes 12, 10, 10, 9, 5, 5, and 3, respectively (Figure 4C; Supplementary Figure S9). It was shown that the KOG annotation of *O. unilateralis sensu* lato is incomplete.

#### 3.2.3 Additional annotation

**Figure 5:**
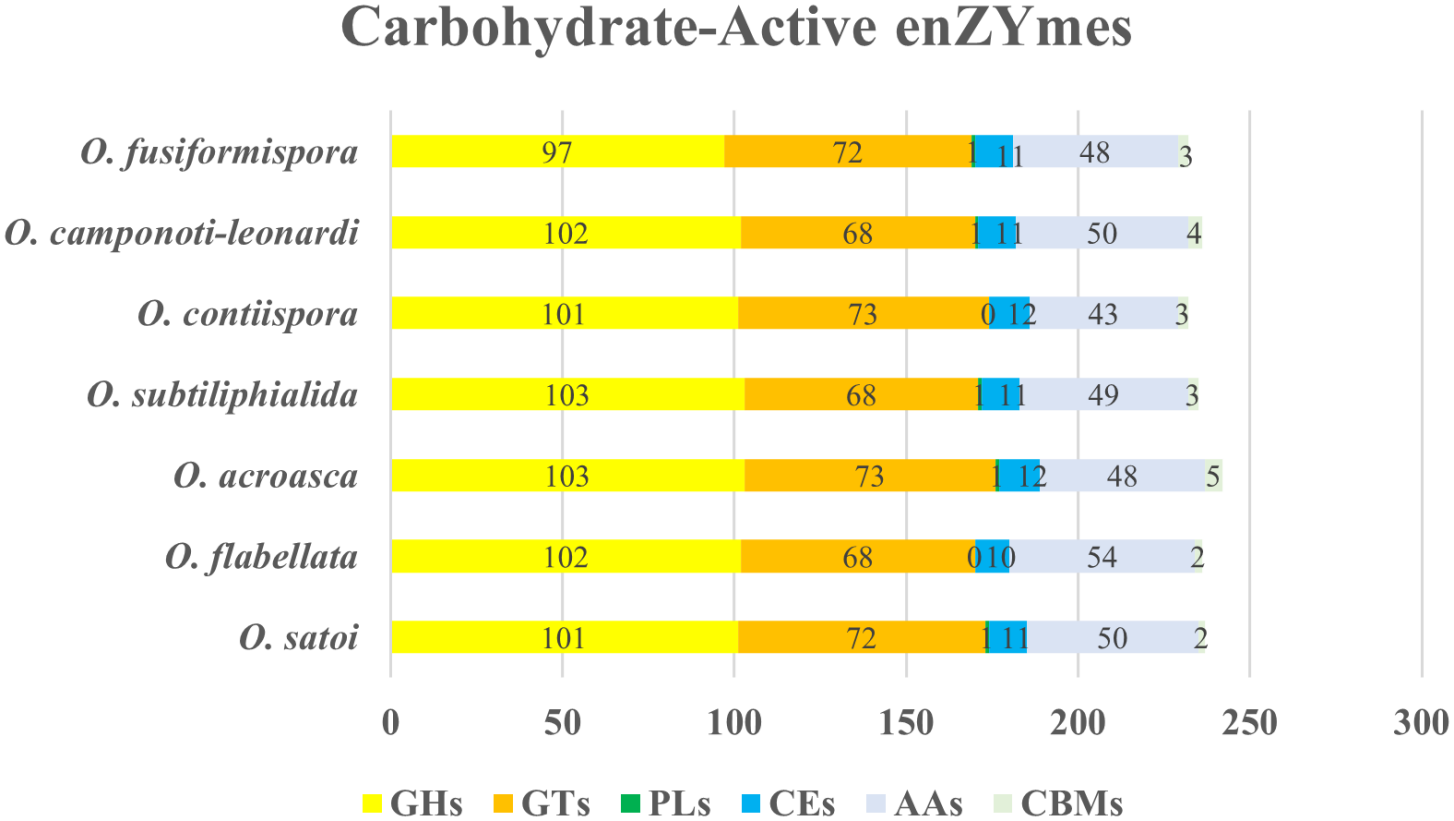
CAZy functional classification chart of seven species of *O. unilateralis sensu* lato. Values represent the number of genes.

The enzyme that played an important role in carbohydrate modification, biosynthesis and degradation of fungi was carbohydrate-active enzymes (CAZy) (Garron and Henrissat, 2019), which was a database of carbohydrate-active enzymes and a special database of carbohydrate enzymes (Drula et al., 2022). Analysis all showed 1,650 genes encoding CAZy in 7 species genomes of *O. unilateralis* complex. Among them, glycoside hydrolases (GHs), glycosyl transferases (GTs) and auxiliary activities (AAs) were the three most abundant enzymes, accounting for 41.81–43.83%, 28.81–31.47% and 18.53–21.88%, respectively, (Figure 5). Therefore, the 7 species of *O. unilateralis sensu* lato should possibly breakdown complex carbohydrates and capture more energy.

### 3.3 Basic characteristics of twelve genomes of *Ophiocordyceps unilateralis* complex

The genome of *O. fusiformispora* was characterized and compared with its 11 related taxa (Supplementary Table S4). *O. satoi* was the largest (75.05 Mb), followed by *O. fusiformispora* (51.00 Mb), and *O. camponoti-rufipedis* had the smallest genome size (21.90 Mb). The genome containing the highest total genes number was *O. polyrhachis-furcata* (10146), while *O. subtiliphialida* had the lowest total genes number (6700). However, the GC contents of *O. unilateralis*, *O. subtiliphialida*, *O. satoi*, *O. polyrhachis-furcata*, *O. fusiformispora*, *O. flabellata*, *O. contiispora*, *O. camponoti-saundersi*, *O. camponoti-rufipedis*, *O. camponoti-leonardi*, *O. camponoti-floridani*, and *O. acroasca* were 55.5, 53.22, 44.05, 43.3, 41.81, 44.77, 46.67, 40.00, 56.10, 45.88, 48.41, and 50.11%, respectively. The results showed that closely related species had similar genome sizes and GC contents.

### 3.4 Analysis of secondary metabolite biosynthesis gene cluster

#### 3.4.1 Overview of twelve genomic BGCs of *Ophiocordyceps unilateralis* complex

AntiSMASH and local BLAST analyses showed that *O. fusiformispora* possessed 29 SM BGCs, including 7 NRPSs, 6 PKSs, 5 Other, 3 Terpene, 2 hybrids NRPS + Other, 1 NRPS-like, 1 T3PKS, Hybrids PKS + NRPS (Supplementary Tables S5, S6). The genome of *O. fusiformispora* had 6 PKSs, including 3 highly reducing (HR) PKSs and 3 non-reducing (NR) PKSs. Only 34.48% of these BGCs indicated gene homologies with known clusters in the MIBiG database.

By further comparison with the gene sequences of other 11 species of *O. unilateralis sensu* lato, the results showed that *O. contiispora*, *O. subtiliphialida*, *O. camponoti-rufipedis*, *O. camponoti-floridani*, *O. satoi*, *O. polyrhachis-furcata*, *O. flabellata*, *O. camponoti-saundersi*, *O. acroasca*, *O. camponoti-leonardi*, *O. fusiformispora*, and *O. unilateralis* had 27, 25, 26, 29, 28, 28, 32, 36, 25, 36, 29, 26, and 26 putative SM BGCs, respectively. The putative SM BGCs presumably contained the number of NRPS were small difference (6–8). In the analysis of 12 genomes in *O. unilateralis* complex species, only *O. camponoti-floridani* (1), *O. camponoti-saundersi* (3), *O. fusiformispora* (1), and *O. unilaterali*s (2) had putative hybrid NRPS-like gene. There was a large difference in the number of Terpene (3–8), but only *O. polyrhachis-furcata*, *O. acroasca*, *and O. unilateralis* had no T3PKS gene. *O. contiispora* and *O. flabellata* had 2 putative hybrids PKS-NRPS, and each of the remaining 10 *O. unilateralis* complex species had only 1 putative hybrids PKS-NRPS. The SM BGCs presumably contained the highest number of *O. camponoti-rufipedis* hybrids NRPS-Other (6), followed by *O. satoi* (4), *O. acroasca* (3), *O. camponoti-saundersi* (3), *O. subtiliphialida* (2), *O. flabellata* (2), *O. camponoti-leonardi* (2), *O. fusiformispora* (2), *O. unilateralis* (2), *O. contiispora* (1), *O. camponoti-floridani* (1), *and O. polyrhachis-furcata* (1). Of the 12 genome-predicted putative SM BGCs, only *O. satoi* and *O. fusiformispora* had 1 hybrids PKS-NRPS-Other, and only *O. camponoti-leonardi* had putative hybrids PKS-Other. In addition, antiSMASH and local BLAST analyses of 12 species of *O. unilateralis* complex also revealed unequal Other gene, with *O. fusiformispora* as the largest (5), followed by *O. camponoti-floridani* (2), *O. satoi* (2), *O. polyrhachis-furcata* (2), *O. flabellata* (2), *O. camponoti-saundersi* (2), *O. unilateralis* (2), *O. contiispora* (1), *O. camponoti-leonardi* (1), and *O. subtiliphialida* (1). Furthermore, only *O. camponoti-rufipedis* and *O. acroasca* had no Other genes. It could be seen that the number and type of putative SM BGCs varied considerably between different species within the same genus.

The 12 species in *O. unilateralis sensu* lato differ greatly in the amount containing putative PKS between 5 and 15. Among the putative PKS, *O. camponoti-saundersi* (15) had the highest number, followed by *O. flabellata* (14) and *O. camponoti-leonardi* (14), with *O. camponoti-rufipedis* (5) had the least. The *O. camponoti-saundersi* genome had 15 PKSs, including 10 HR-PKSs and 4 NR-PKSs. It had been shown that the No of cluster and type in the putative SM BGCs of closely related species were similar. For example, *O. camponoti-leonardi*, *O. flabellata*, *O. polyrhachis-furcata*, and *O. camponoti-saundersi* were sister species with a number of PKS between 13 and 14.

#### 3.4.2 Difference analysis of twelve genomic domains in *Ophiocordyceps unilateralis sensu* lato

Genome mining results for 12 species of the *O. unilateralis* complex showed that, in addition to differences in the number and types of putative SM BGCs in different species of the same genus, there were also significant differences in the domains of NRPS, PKS, and hybrid PKS-NRPS (Supplementary Table S5). The domains shared by all 12 genomes were KS-AT-DH-MT-ER-KR-ACP and SAT-KS-AT-PT-ACP-ACP-Te. With the exception of *O. contiispora*, the remaining 11 genomes had SAT-KS-AT-PT-ACP-ACP-ACP-Te domain. Only *O. satoi* and *O. fusiformispora* had Hybrid PKS-NRPS-Other of the KS-AT-DH-MT-KR-P-C-A-P-Te-MFS-Dehydrogenase-CYP-MT domain. It had been hypothesized that differences in key enzyme domains might be caused by kinship.

Each of species had unique domain. The A-A-A-P-C-A-P-C was the domain unique to *O. fusiformispora*, while the domains specific of *O. satoi* were P-A-C-A-P and A-P-Te-MT-CYP. The unique structural domains of *O. contiispora* were A-P-A-P-C-A-P-C and KS-AT-DH-KR-ACP-4CL. The A-A-P-C-P-C, AT-DH-MT-ER-KR-ACP, AspB-A-MT-Te-HutI, and KS-AT-PT-ACP-Te were the domains specific to *O. flabellata*. *O. acroasca* had special domains of P-C-A-P-C, C-A-MT-Te-HutI, A-P-KS-AT-ER-ACP-Te, KS-AT-PT-ACP-Te. The A-P-C-P-C-A-C-P-C-P-C, and A-C-A-P were the unique domains to *O. subtiliphialida*. The unique structural domains of *O. camponoti-rufipedis* were P-A and KS-AT-KR-P-C-A-P-Te, while the C-A-P-A-P-A-A-P-C, P-Te, C-A, ER-KR-ACP, KS-AT-Cya-ER-KR-ACP, SAT-KS-AT-PT-ACP-MTH-MT, and SAT-KS-AT-PT-ACP-Aes were the domains of *O. camponoti-leonardi*. The A-P-C-A-P-C, C-A-P-C-A-P-A-C-A-P-C, C-A-P-Te, KS-AT-DH-MT-KR, KS-AT-DH-MT-ER-KR, and KS-AT-PT-ACP-HTH-MT were the unusual domains of *O. unilateralis*, while the individual domains to *O. camponoti-saundersi* were A-C-A-P-C-P-C and A-A-P-C-A-P-C. *O. camponoti-floridani* was characterized by the C-A-Te. The KS-AT-MT-KR-P-C-A-P-Te, PT-KS-AT-DH-MT-ER-KR-ACP, SAT-KS-AT-PT-ACP-ACP, SAT-KS-AT-PT were the domains specific to *O. polyrhachis-furcata*.

Furthermore, there were many domains that only a few species had or that a few species did not have. For example, only *O. camponoti-rufipedis* and *O. camponoti-floridani* had the C-A-P-A-P-A-P-C domain. Except for *O. contiispora*, *O. satoi*, *O. camponoti-rufipedis*, and *O. camponoti-floridani*, all the other genomes had the A-P-Te domain. Only *O. flabellata*, *O. contiispora*, *O. camponoti-saundersi*, *and O. unilateralis* existed the KS-AT-DH-MT-KR-ACP-C-A-P-Te domain, while the KS-AT-DH-ER-KR-ACP existed only in the genomes of *O. polyrhachis-furcata*, *O. flabellata*, *O. camponoti-leonardi*, *O. unilateralis*, *O. camponoti-saundersi*, and *O. camponoti-floridani*.

#### 3.4.3 Analysis of SM BGCs for twelve species of *Ophiocordyceps unilateralis* complex

**Figure 6:**
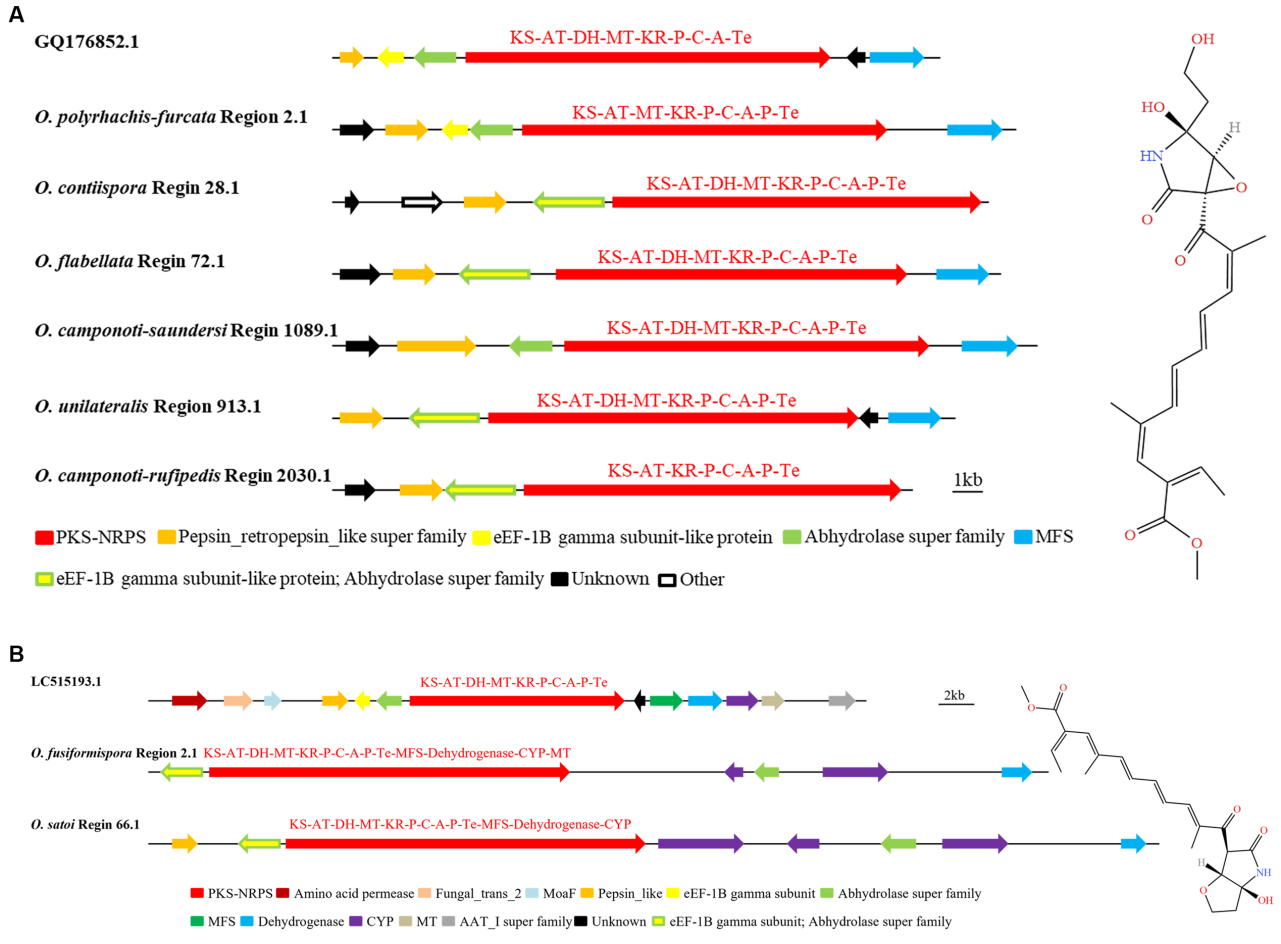
Comparison of putative BGC of NG-391 **(A)** and lucilactaene **(B)** in *O. unilateralis* complex. The number after the region and the number before the decimal point represent the scaffold, and the number after the decimal point represents the gene cluster.

NG-391 were 7-desmethyl analogs of fusarin C (Donzelli et al., 2010). GQ176852.1 was a biosynthetic gene cluster that produced the SM NG-391. It was found that the whole genome sequences of the six species in the *O. unilateralis sensu* lato had homologous regions that were highly similar to the sequence of the NG-391 gene in MIBiG, with up to 100% similarity. They were *O. polyrhachis-furcata* Region 2.1, *O. contiispora* Region 28.1, *O. flabellata* Region 72.1, *O. camponoti-saundersi* Region 1089.1, *O. unilateralis* Region 913.1, and *O. camponoti-rufipedis* Region 2030.1 (Figure 6A). In addition to the key enzyme hybrid PKS-NRPS (KS-AT-DH-MT-KR-P-C-A-Te), NG-391 production also required the modified gene of Pepsin_retropepsin_like super family, eEF-1B gamma subunit-like protein and Abhydrolase super family, MFS, and unknown functional genes to co catalyze synthesis. The domain of Hybrid PKS-NRPS in *O. polyrhachis-furcata* Region 2.1, *O. contiispora* Region 28.1, *O. flabellata* Region 72.1, *O. camponoti-saundersi* Region 1089.1, *O. unilateralis* Region 913.1, and *O. camponoti-rufipedis* Region 2030.1 was basically KS-AT-DH-MT-KR-P-C-A-P-Te. Compared with known gene clusters, there was one more P in the above species domain, and species *O. polyrhachis-furcata* Region 2.1 had no DH domain, and *O. camponoti-rufipedis* Region 2030.1 had no DH and MT domain. *O. contiispora* Region 28.1 and *O. camponoti-rufipedis* Region 2030.1 had no MFS modified genes. Species *O. contiispora* Region 28.1, *O. flabellata* Region 72.1, *O. unilateralis* Region 913.1, and *O. camponoti-rufipedis* Region 2030.1 shared the genetic sequence of the modified gene eEF-1B gamma subunit-like protein with the Abhydrolase superfamily. This suggested that there were differences between different species of *O. unilateralis* complex in the sequence of genes that made the same compound. It can be seen that there were differences in the gene sequences that predicted the production of the same compound among the different species of the *O. unilateralis sensu* lato, which mainly manifest themselves as gene chimerism.

Research showed that the same BGC biosynthesizes lucilactaene and NG-391 due to their structural similarity. The chemical structure of lucilactaene also was similar to fusarin C (Wiebe and Bjeldanes, 1981; Kato et al., 2020). The BGC (GenBank: LC515193.1) for the catalytic synthesis of lucilactaene were hybrid PKS-NRPS (KS-AT-DH-MT-KR-P-C-A-P-Te), amino acid permease and fungal_trans_2, moaF, pepsin_like, eF-1B gamma subunit, abhydrolase super family, MFS, dehydrogenase, CYP, MT, and AAT_I super family (Figure 6B). It was found that *O. fusiformispora* Region 2.1 and *O. satoi* Regin 66.1 were highly similar to LC515193.1. However, the modified genes MFS, dehydrogenase, CYP and MT, which catalyzed the synthesis of lucilactaene, were chimerized with the hybrid PKS-NRPS. And the modified gene eEF-1B gamma subunit and abhydrolase super family were also chimed together. *O. fusiformispora* Region 2.1 and *O. satoi* Regin 66.1 had no amino acid permease, fungal_trans_2 and AAT_I super family modification genes. Based on phylogenetic relationships, *O. fusiformispora* and *O. satoi* were in a clade. It was speculated that in the process of evolution, the more closely related species had the same trend of gene change.

**Figure 7:**
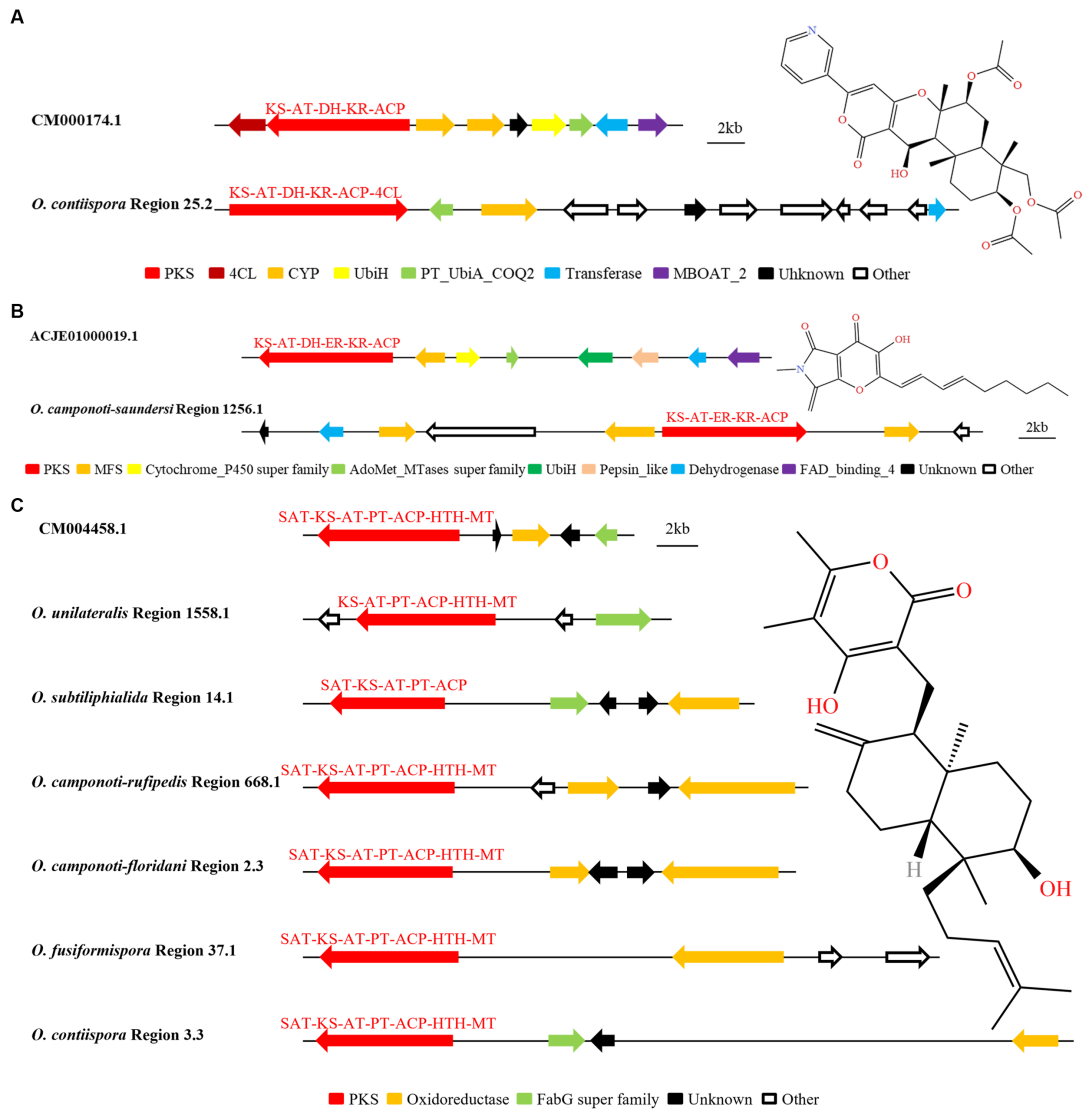
Comparison of biosynthesis of putative pyripyropene A **(A)**, pyranonigrin E **(B)** and higginsianin B **(C)**. The number after the region and the number before the decimal point represent the scaffold, and the number after the decimal point represents the gene cluster.

Through AntiSMASH and local BLAST analysis comparison, it was found that *O. contiispora* Region 25.2 and *O. camponoti-saundersi* Region 1256.1 had hypothetical BGC similar to the catalytic synthesis of pyripropene A and pyranotigrin E compounds, respectively. The SM BGC (GenBank: CM000174.1) that produced pyripyropene A were PR-PKS (KS-AT-DH-KR-ACP), two CYPs, UbiH, Transferase, MBOAT_2, and unknown functional genes (Itoh et al., 2010). It was found that *O. contiispora* Region 25.2 was 68% similar to the known gene cluster CM000174.1 (Figure 7A). However, *O. contiispora* Region 25.2 did not contain the UbiH and MBOAT_2 modification genes necessary to produce the appropriate A, and the 4CL and PKS genes necessary for the production of this compound were chimerized together. It had been speculated that the apparent gene fusion in *O. contiispora* Region 25.2 was an adaptation of the evolutionary process. According to MIBiG database, HR-PKS (KS-AT-DH-ER-KR-ACP), Pepsin_like, Cytochrome_P450 super family, AdoMet_MTases super family, UbiH, MFS, Dehydrogenase and FAD_binding_4 were the BGC for the catalytic synthesis of pyranonigrin E (GenBank: ACJE0100009.1). AntiSMASH and local BLAST analysis showed that the similarity of *O. camponoti-saundersi* Region 1256.1 was up to 100% compared with the BGC of catalytic synthesis of pyranonigrin E (Figure 7B). However, the domain of the PKS of *O. camponoti-saundersi* Region 1256.1 was KS-AT-ER-KR-ACP. The modified genes were dehydrogenase, three MFS, an unknown functional gene, and an Other gene. It could be seen that *O. camponoti-saundersi* Region 1256.1 had obvious gene loss in species evolution.

The analysis found that *O. unilateralis* Region 1558.1, *O. subtiliphialida* Region 14.1, *O. camponoti-rufipedis* Region 668.1, *O. camponoti-floridani* Region 2.3, *O. fusiformispora* Region 37.1, and *O. contiispora* Region 3.3 were highly similar to CM004458.1 for the catalytic synthesis of higginsianin B (Figure 7C). The remaining species did not have the potential to synthesize higginsianin B. *O. unilateralis* Region 1558.1 did not contain SAT domain and oxidoreductase modified genes. *O. fusiformispora* Region 37.1, *O. camponoti-leonardi* Region 668.1 and *O. camponoti-floridani* Region 2.3 did not have fabG super family, and O. subtiliphialida Region 14.1 did not have HTH and MT domains. The BGC similarity in the synthesis of certain compounds between *O. unilateralis* complex species was very high. It was speculated that there was a certain level of horizontal gene transfer between these species, and the direction and position of these gene sequences were variable, so gene chimerism, gene loss or addition might occur between different species.

### 3.5 Cluster analysis

#### 3.5.1 SM BGCs cluster analysis

**Figure 8:**
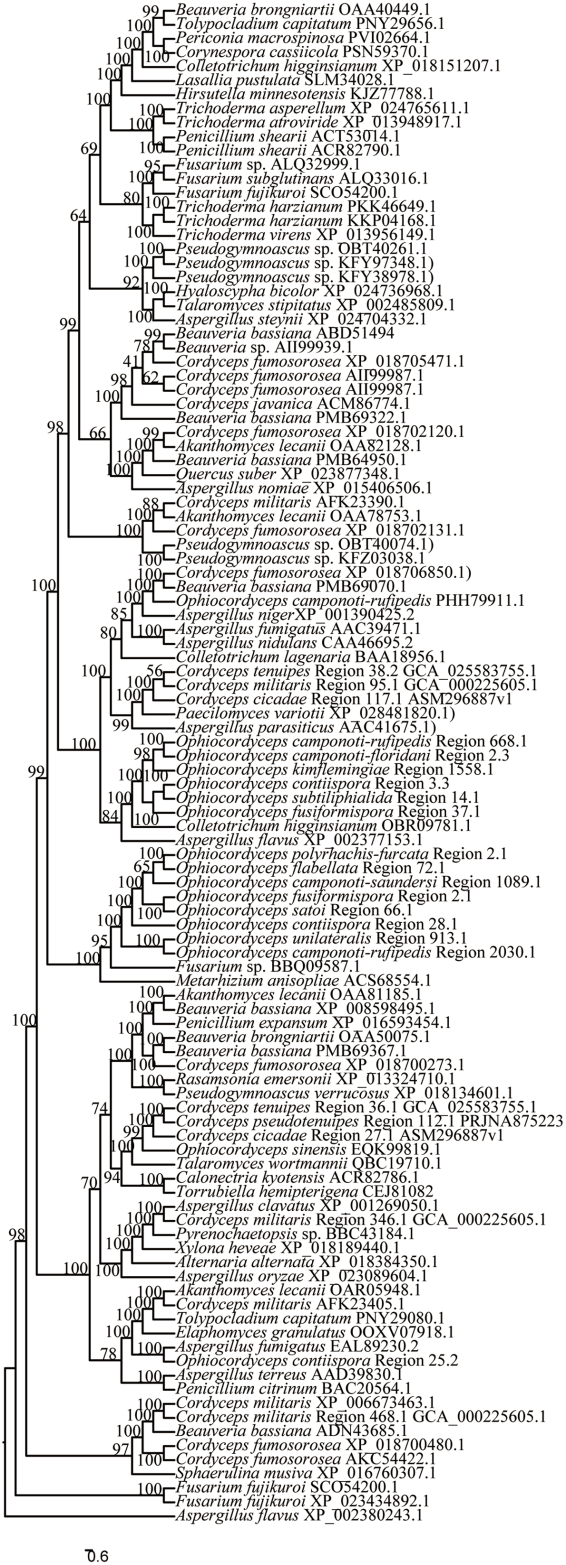
Clustering tree of PKS or hybrid PKS-NRPS proteins between *O. unilateralis* complex species and other fungi. Values at the nodes represent bootstrap values. The scale bar 0.6 indicates the number of expected mutations per site.

The clustering results of *O. unilateralis sensu* lato PKS and hybrid PKS-NRPS proteins with other fungal PKS and hybrid PKS-NRPS proteins showed that *O. unilateralis* Region 1558.1, *O. subtiliphialida* Region 14.1, *O. camponoti-rufipedis* Region 668.1, *O. camponoti-floridani* Region 2.3, *O. fusiformispora* Region 37.1, and *O. contiispora* Region 3.3 clustered on a separate branch from *Colletotrichum higginsianum* (OBR09781.1), which catalyzed higginsianin B biosynthesis, and the six regions presumably catalyze the biosynthesis of higginsianin B or its analogs (Figure 8). *O. contiispora* Region 25.2 clustered with the PKS protein and possibly catalyzes pyripyropene A synthesis in *Aspergillus fumigatus* (EAL89230.2) (Figure 8). Moreover, *O. contiispora* Region 25.2 might catalyze the biosynthesis of pyripyropene A or its analogs. *O. polyrhachis-furcata* Region 2.1, *O. contiispora* Region 28.1, *O. flabellata* Region 72.1, *O. camponoti-saundersi* Region 1089.1, *O. unilateralis* Region 913.1, and *O. camponoti-rufipedis* Region 2030.1 clustered with *Metarhizium anisopliae* (ACS68554.1), which catalyzed the biosynthesis of NG-391 or its analogs (Figure 8). Likewise, *O. fusiformispora* Region 2.1 and *O. satoi* Regin 66.1 clustered with *Fusarium* sp. (BBQ09587.1) catalyzed lucilactaene synthesis and probably produced lucilactaene or its analogs (Figure 8).

#### 3.5.2 Comparative analysis of NR-PKS homologous region cluster tree and multi-gene genetic distance tree

**Figure 9:**
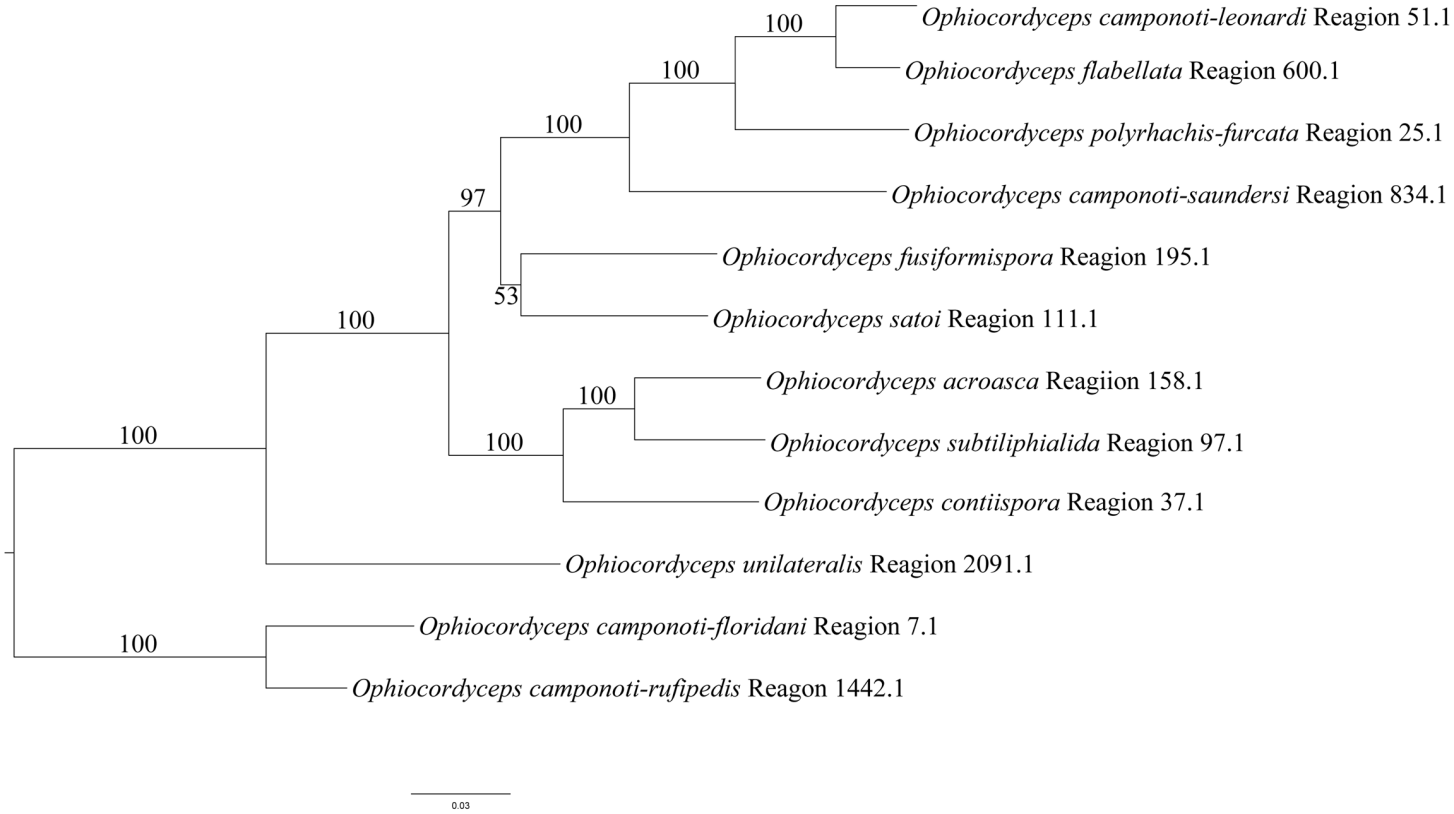
Clustering tree of the *O. unilateralis* complex species were constructed by the NR-PKS gene dataset using the Maximum likelihood (ML) analysis. Values at the nodes represent bootstrap values. The scale bar 0.03 indicates the number of expected mutations per site.

According to the NR-PKS (SAT-KS-AT-PT-ACP-ACP-Te) clustering tree results of *O. unilateralis sensu* lato species (Figure 9), the 12 species in this study form independent branches each, and receive high support, almost 100%. Similar to the results of phylogenetic tree of *O. unilateralis* complex constructed from the five-gene dataset (Figure 2), *O. acroasca*, *O. subtiliphialida*, and *O. contiispora* were the most closely related species. The results of five-gene phylogenetic tree showed that *O. fusiformispora* and *O. satoi* were sister species, but *O. fusiformispora* and *O. satoi* had low bootstrap values in NR-PKS clustering tree. Although the results of the NR-PKS cluster tree were similar to those of the phylogenetic tree constructed with five genes, they were not fully applicable to molecular phylogenetic studies of species and could provide further evidence for phylogenetic studies of species.

## 4 Discussion

Many phylogenetic classifications of the *O. unilateralis* complex had been undertaken, and these groups had been continuously supplemented and improved on the basis of molecular phylogeny, morphology and ecology (Evans et al., 2011b; Kobmoo et al., 2012, 2015; Araújo et al., 2018; Wei et al., 2020). As the same time, the use of multi-gene combined datasets for species classification had been widely adopted. In this study, five genes dataset of *O. fusiformispora* (SSU, LSU, TEF, *RPB1* and *RPB2*) were collected and placed into 124 taxa of known species of *Ophiocordyceps*. The phylogenetic framework of *Ophiocordyceps* was reconstructed, and the four clades of *Ophiocordyceps* was obtained with strong support rates, namely *Hirsutella* clade, *O. sphecocephala* clade, *O. sobolifera* clade, and *O. ravenelii* clade. The phylogenetic tree indicated that *O. fusiformispora* was clustered in the core clade of the *O. unilateralis* complex (Figure 2). *O. fusiformispora*, fromed a lineage with *O. bifertilis* and *O. satoi*, as well as there hosts were all *Polyrhachis* sp. This phylogenetic framework was consistent with the findings of Tang et al. (2023a,b).

In this study, this study presented the basic genomic characteristics of 12 species of *O. unilateralis sensu* lato, the results showed that genome size of the 12 species in *O. unilateralis* complex ranged from 21.90 to 75.05 Mb, with the GC content of 40.00 to 56.10%, the N50 of 11,763 to 3,776,764 bp, and the total number of genes of 6,700 to 10,146. The genome level of *O. unilateralis sensu* lato was different for different species. The more closely related species had smaller differences in genome size, GC content, N50, and total number of genes, and the more closely related species were likely to perform some similar biological functions in the environment. The BGCs of SMs that might be associated with ant manipulation behavior were analyzed through a gene mining analysis of 12 species of the *O. unilateralis* complex. The number of putative SM BGCs in the genomes of the 12 species of the *O. unilateralis sensu* lato ranged from 25 to 36. Interestingly, the more closely related the species, the more similar the putative number of SM BGCs. In general, the number and types of SM BGCs obtained for the 12 species of *O. unilateralis sensu* lato were different, and for the closely related species they were more similar. Similar results were obtained that the sister species of *Cordyceps*, *C. pseudotenuipes* H. Yu, Q. Y. Dong, and Y. Wang and *C. tenuipes* (Peck) Kepler, B. Shrestha, and Spatafora, had the potential to synthesize dimethylcoprogen, epichloenin A, wortmanamide A/B, beauvericin, etc. (Lu et al., 2022). The number of NPRS-like, PR-PKS, Hybrid PKS-NRPS-Other and Hybrid PKS-Other species in each species was small or even absent. It was possible that for most species of *O. unilateralis sensu* lato, these types were not necessary for their life activities. The T3PKS and Other genes were present in most species. For some species, such as *O. camponoti-rufipedis*, *O. acroasca*, *O. polyrhachis-furcata* and *O. unilateralis*, were missin. It was also unknown what functions the existing *O. unilateralis sensu* lato species might perform in their life activities.

Yuan et al. (2019) found likely existing NRPS and PKS in the genome of *C. militaris* by genome mining. In this study, many putative NRPS, PKS, and hybrid NRPS-PKS were obtained. Many of the compounds were more similar to known gene clusters, such as clvaric acid, squalestatin S1, terpendole E, cichorine, YWA1, 1-heptadecene, alternariol, naphthopyrone, ferrichrome, phenalamide, ajudazol A, pellasoren, and epoxy-Janthitreys could not be analyzed further because there were no modified genes, known gene clusters, key enzymes, or indoles or terpenes. The unique NR-PKS domains of *O. unilateralis sensu* lato, such as SAT-KS-AT-PT-ACP-MTH-MT and SAT-KS-AT-PT-ACP-Aes of *O. camponoti-leonardi*, and KS-AT-PT-ACP-Te of *O. flabellata*, *O. polyrhachis-furcata* KS-AT-MT-KR-P-C-A-P-Te and 13 species all had the common domain HR-PKS (KS-AT-DH-MT-ER-KR-ACP), and the unique and common domain played an important role in the life cycle of each species. Catalyzed the corresponding compounds to perform biological functions, reflecting the genetic differences between species.

By comparing the transcriptomes, Will et al. (2020) pointed out that fusarin C might act as a virulence factor to alter ant behavior. Song et al. (2004) identified and established that the fusarin C backbone was biosynthesized by a hybrid PKS-NRPS enzyme. Niehaus et al. (2013) found the fusarin C BGC consisted of nine co-expressed genes, and only four genes were importance to catalyzed synthesized fusarin C. NG-391 was a derivative of fusarin C, which was 7 ‘-demethylated and had a similar hybrid PKS-NRPS biosynthesis mechanism to fusarin C, with the hybrid PKS-NRPS gene *NGS1* involved in the synthesis of NG-391 compounds (Wiebe and Bjeldanes, 1981; Gelderblom et al., 1983; Barrero et al., 1991; Donzelli et al., 2010). In this study, the whole genome sequences of 6 species in *O. unilateralis sensu* lato (*O. polyrhachis-furcata* Region 2.1, *O. contiispora* Region 28, *O. flabellata* Region 72.1, *O. camponoti-saundersi* Region 1089.1, *O. camponoti-rufipedis* Region 2030.1, *O. unilateralis* Region 913.1) were present in homologous regions as high as 100% similar to the NG-391 gene sequence produced in MIBiG database. The genomes of *O. kimflemingiae* and *O. camponoti-floridani* in *O. unilateralis sensu* lato had homologous regions of gene clusters necessary for fusarin C synthesis (De Bekker et al., 2015; Wichadakul et al., 2015). Therefore, the6 species of *O. unilateralis sensu* lato might produce NG-391 compounds similar to fusarin C and might manipulate ant behavior as virulence factors. Lucilataene had the same BGC as NG-391 and was also a derivative of fusarin C (Kato et al., 2020). It was found that *O. fusiformispora* and *O. satoi* had a homologous region that was highly similar to the lucilactaene gene sequence (GenBank: LC515193.1), which maight produce lucilactaene or its analogs. Kato et al. (2020) showed that lucilataene from strain *Fusarium* sp. RK 97–94 showed strong antimalarial activity. Species of the *O. unilateralis sensu* lato maight produce lucilactaene or its analogs in the process of infecting the host ant, thus inhibiting the normal operation of the ant’s associated cell cycle, resulting in a disease-like state of the ant. At the same time, it could also be seen that different species produced the same compound, such as NG-391 or lucilactaene, there were differences in gene sequences of *O. unilateralis sensu* lato, mainly manifested as gene mosaic and deletion.

Omura et al. (1993) isolated the active compound pyripyropene A (PPPA) from the soil fungus *A. fumigatus* FO-1289. PPPA, an acyl-CoA, was a selective inhibitor of cholesterol acyltransferase 2-selective inhibitor (ACAT2), the selective inhibition of ACAT2 in intestine and liver could effectively combat atherosclerosis. Alleviation of hypercholesterolemia and atherosclerosis was demonstrated in a mouse model of hyperlipidemia. The putative BGC associated with the catalytic synthesis of pyripyropene A compound (GenBank: CM000174.1) existed in Region 25.2 of *O. contiispora*, and its homologous region was similar to CM000174.1 was 68%. Pyripyropene A might be synthesized in *O. contiispora* Region 25.2. The function of pyripyropene A in the O*. unilateralis sensu* lato required further investigation. The higginsianin B was a C-8 isomer of the diperpenoid BR-050. The higginsianin B was found to have good anti-proliferation and cell-blocking activity against six cancer cell lines, but no cytotoxic activity. The compound could be further studied as a potential cancer drug (Cimmino et al., 2016). The study found that *O. unilateralis* Region 1558.1, *O. subtiliphialida* Region 14.1, *O. camponoti-rufipedis* Region 668.1, *O. camponoti-floridani* Region 2.3, *O. fusiformispora* Region 37.1 and *O. contiispora* Region 3.3 might catalyze the synthesis of higginsianin B or its analogs, which might inhibit some cells in the life activities of ants during the infection of *O. unilateralis sensu* lato species, thus affecting the normal life activities of ants and causing the appearance of disease. The BGC similarity of synthesis of certain compounds between *O. unilateralis* complex species was very high. The pyranonigrin E was isolated from the marine fungus *A. niger* and was a metabolite of the hybrid PKS-NRPS (Awakawa et al., 2013). It was found that of the 12 genomes of *O. unilateralis sensu* lato, only the Region 1256.1 of *O. camponoti-saundersi* had homologous regions that were up to 100% similar to the catalytic production of pyranonigrin E BGC. The HR-PKS protein sequence of *O. camponoti-saundersi* Region 1256.1 and the HR-PKS protein sequence of pyranonigrin E catalyzed synthesis (GenBank: EHA19289.1) could not be clustered together, thus clustering analysis was not performed. The Region 1256.1 of *O. camponoti-saundersi* showed clear gene loss during evolution, for example the HR-PKS protein sequence had lost its DH domain and therefore could not be clustered with EHA19289.1. The *O. unilateralis sensu* lato species was commonly distributed in tropical rainforests, and many studies track its behavior on a one-year basis, with a long growth cycle (Araújo et al., 2018; Cardoso et al., 2019), For *O. unilateralis sensu* lato species distributed in tropical rainforests, the production of pyranonigrin E, a compound that cleaned up superoxide free radicals in their bodies in time, might be the reason for their longer growth time.

In this study, five compounds including NG-391, lucilactaene, higginsianin B, pyripyropene A and pyranonigrin E were discovered from the 12 species of *O. unilateralis sensu* lato. These compounds might have their own or similar biological properties in each species. The clusters of genes catalyzed by different species to synthesize the same compound were different. It was conjectured that there was some degree of horizontal gene transfer among these species, that the direction and location of these gene sequences was variable, and that gene mosaicism, gene loss, or addition may occur among different species. Different species of *O. unilateralis sensu* lato might had evolved to adapt to different environmental pressures. Clustering trees based on NR-PKS genes had been constructed for the *O. unilateralis sensu* lato, and the results were similar to those obtained using phylogenetic trees constructed from a five-gene dataset. The topological structure of *Ophiocordyceps* was consistent with that of Tang et al. (2023a,b) by constructing five genes. Therefore, the use of NR-PKS to construct cluster trees may provide further evidence for the construction of phylogenetic trees in future studies, but it is not yet fully applicable to the study of molecular systematics of species.

## 5 Conclusion

Twelve species showed significant differences in genome size, GC content, number and type of putative SM BGC, NPPS domain, PKS domain and Hybrid PKS-NRPS domain between different species of the same genus. The genome-wide basic characteristics of the 12 species revealed that closely related species had similar genome sizes and GC contents.

Each species had its own distinct domain. The A-A-A-P-C-A-P-C domain was endemic to *O. fusiformispora*, and its sister species, *O. satoi*, was endemic to P-A-C-A-P and A-P-Te-MT-CYP. The KS-AT-DH-MT-ER-KR-ACP and SAT-KS-AT-PT-ACP-ACP-Te domains were present in all 12 genomes. In particular, the ML trees and BI trees with SAT-KS-AT-PT-ACP-ACP-Te homologous regions were highly similar to the polygenic distance trees.

A putative BGC of five compounds, NG-391, lucilactaene, higginsianin B, pyripyropene A and pyranonigrin E was excavated. NG-391 and lucilactaene were 7-desmethyl analogs of fusarin C. In addition, five putative SM BGCs were found to be highly similar to known gene clusters. The BGC similarity in the synthesis of certain compounds between species of the *O. unilateralis* comple was very high. It was speculated that there was some level of horizontal gene transfer between these species, and that the direction and location of these gene sequences were variable, so that gene chimerism, gene loss, or addition might occur between different species.

## Data availability statement

The original contributions presented in the study are publicly available. This data can be found here: NCBI - GCA_002591395.1, GCA_012980515.1, GCA_001633055.2, GCA_003339415.1, and GCA_001272575.2.

## Author contributions

YL: Conceptualization, Data curation, Formal analysis, Investigation, Methodology, Project administration, Resources, Software, Supervision, Validation, Visualization, Writing – original draft. DT: Data curation, Formal analysis, Resources, Software, Writing – review & editing. ZL: Data curation, Formal analysis, Resources, Software, Writing – review & editing. YC: Data curation, Software, Writing – review & editing. JM: Data curation, Software, Writing – review & editing. LL: Data curation, Software, Writing – review & editing. HY: Investigation, Funding acquisition, Supervision, Writing – review & editing. JZ: Data curation, Resources, Software, Writing - review & editing.

## Footnotes

^1^ https://www.ncbi.nlm.nih.gov/

^2^ https://antismash.secondarymetabolites.org/

^3^ www.softberry.com/

^4^ https://nrps.igs.umaryland.edu/

^5^ https://blast.ncbi.nlm.nih.gov/

^6^ http://iqtree.cibiv.univie.ac.at/
