# Supplementary material for: Genomic comparative analysis of Ophiocordyceps unilateralis sensu lato
Source: Front Microbiol. 2024 Apr 15;15:1293077. doi: 10.3389/fmicb.2024.1293077 (PMC11057048; doi:10.3389/fmicb.2024.1293077)
Supplement: Supplementary file 1 [file Data_Sheet_1.docx]

**Genomic comparative analysis of *Ophiocordyceps unilateralis* sensu lato**

Yingling Lu^1, 2, #^, Dexiang Tang^1, 2, #^, Zuoheng Liu^1, 2, #^, Jing Zhao^1, 2^, Yue Chen^1, 2^, Jinmei Ma^1, 2^, Lijun Luo^1, 2^ and Hong Yu^1, 2,^ *

^1^ Yunnan Herbal Laboratory, College of Ecology and Environmental Sciences, Yunnan University, Kunming, Yunnan 650504, China; lyinglingua@163.com (Y.L.); TangDX1516@163.com (D.T.); 2416338332@qq.com (Z.L.); zhaojing@mail.ynu.edu.cn (J.Z.); cy106daytoy@163.com (Y.C.); [1202230164@mail.ynu.edu.cn](mailto:1202230164@mail.ynu.edu.cn) (J.M.); 1211918264@qq.com (L.L.); hongyu@ynu.edu.cn (H.Y)

^2^ The International Joint Research Center for Sustainable Utilization of Cordyceps Bioresources in China and Southeast Asia, Yunnan University, Kunming 650091, China

^*^ Correspondence: hongyu@ynu.edu.cn (H.Y); Tel.: 13700676633

^#^ These authors contributed equally to this work.

Table S1. Specimen information and GenBank accession numbers for taxa used in the phylogenetic tree.

| **Species name** | **Voucher information** | **Host** | **SSU** | **LSU** | ***TEF1α*** | ***RPB1*** | ***RPB2*** | **Location information** |
| --- | --- | --- | --- | --- | --- | --- | --- | --- |
| *Ophiocordyceps acicularis* | ARSEF 5692 | Coleoptera | DQ522540 | DQ518754 | DQ522322 | DQ522368 | DQ522418 | Korea |
| *Ophiocordyceps acicularis* | OSC 128580 | Coleoptera | DQ522543 | DQ518757 | DQ522326 | DQ522371 | DQ522423 |  |
| ***Ophiocordyceps acroasca*** | **YFCC 9049** | ***Camponotus* sp.** | **ON555837** | **ON555918** | **ON567757** | **ON568677** | **ON568130** | **China** |
| *Ophiocordyceps acroasca* | YFCC 9019 | *Camponotus* sp. | ON555838 | ON555919 | ON567758 | ON568678 | ON568131 | China |
| *Ophiocordyceps acroasca* | YFCC 9016 | *Camponotus* sp. | ON555841 | ON555922 | ON567761 | ON568681 | ON568134 | China |
| *Ophiocordyceps albacongiuae* | RC20 | *Camponotus* sp. | KX713633 |  | KX713670 |  |  | Colombia |
| *Ophiocordyceps aphodii* | ARSEF 5498 | Coleoptera | DQ522541 | DQ518755 | DQ522323 |  | DQ522419 |  |
| *Ophiocordyceps australis* | HUA 186097 | Hymenoptera | KC610786 | KC610765 | KC610735 | KF658662 |  | Colombia |
| *Ophiocordyceps basiasca* | YHH 20191 | *Camponotus* sp. | ON555828 | ON555910 | ON567748 | ON568672 | ON568121 | China |
| *Ophiocordyceps bifertilis* | YFCC 9012 | *Polyrhachis* sp. | ON555843 | ON555923 | ON567763 | ON568143 | ON568135 | China |
| *Ophiocordyceps bifertilis* | YHH 20162 | *Polyrhachis* sp. | ON555844 |  | ON567764 | ON568144 |  | China |
| *Ophiocordyceps bifertilis* | YHH 20163 | *Polyrhachis* sp. | ON555845 | ON555924 | ON567765 | ON568145 | ON568136 | China |
| *Ophiocordyceps bifertilis* | YHH 20164 | *Polyrhachis* sp. | ON555846 |  | ON567766 | ON568146 |  | China |
| *Ophiocordyceps bifertilis* | YFCC 9048 | *Polyrhachis* sp. | ON555847 | ON555925 | ON567767 | ON568147 | ON568137 | China |
| *Ophiocordyceps bifertilis* | YFCC 9013 | *Polyrhachis* sp. | ON555848 | ON555926 | ON567768 | ON568148 | ON568138 | China |
| *Ophiocordyceps blakebarnesii* | MISSOU5 | *Camponotus* sp. | KX713641 | KX713610 | KX713688 | KX713716 |  | USA |
| *Ophiocordyceps blakebarnesii* | MISSOU4 | *Camponotus* sp. | KX713642 | KX713609 | KX713685 | KX713715 |  | USA |
| *Ophiocordyceps brunneipunctata* | OSC 128576 | Coleoptera | DQ522542 | DQ518756 | DQ522324 | DQ522369 | DQ522420 |  |
| *Ophiocordyceps buquetii* | HMAS_199617 | Hymenoptera | KJ878940 | KJ878905 | KJ878985 | KJ879020 |  | China |
| *Ophiocordyceps camponoti-balzani* | G143 | *Camponotus balzani* | KX713658 | KX713595 | KX713690 | KX713705 |  | Brazil |
| *Ophiocordyceps camponoti-balzani* | G104 | *Camponotus balzani* | KX713660 | KX713593 | KX713689 | KX713703 |  | Brazil |
| *Ophiocordyceps camponoti-bispinosi* | OBIS5 | *Camponotus bispinosus* | KX713636 | KX713616 | KX713693 | KX713721 |  | Brazil |
| *Ophiocordyceps camponoti-bispinosi* | OBIS4 | *Camponotus bispinosus* | KX713637 | KX713615 | KX713692 | KX713720 |  | Brazil |
| *Ophiocordyceps camponoti-chartificis* | MF080 | *Camponotus chartifex* | MK874744 |  | MK863824 |  |  | Brazil |
| *Ophiocordyceps camponoti-femorati* | FEMO2 | *Camponotus femoratus* | KX713663 | KX713590 | KX713678 | KX713702 |  | Brazil |
| *Ophiocordyceps camponoti-floridani* | Flo4 | *Camponotus femoratus* | KX713662 | KX713591 |  |  |  | Brazil |
| *Ophiocordyceps camponoti-floridani* | Flx2 | *Camponotus femoratus* |  | KX713592 | KX713674 |  |  | Brazil |
| *Ophiocordyceps camponoti-hippocrepidis* | HIPPOC | *Camponotus hippocrepis* | KX713655 | KX713597 | KX713673 | KX713707 |  | Brazil |
| *Ophiocordyceps camponoti-indiani* | INDI2 | *Camponotus indianus* | KX713654 | KX713598 |  |  |  | Brazil |
| ***Ophiocordyceps camponoti-leonardi*** | **YFCC 9028** | ***Camponotus leonardi*** | **OR676967** | **OR685713** | **0R946331** |  | **PP091966** | **China** |
| *Ophiocordyceps camponoti-leonardi* | C27 | *Camponotus leonardi* |  |  | JN819019 |  |  | Thailand |
| *Ophiocordyceps camponoti-leonardi* | C25 | *Camponotus leonardi* |  |  | JN819029 |  |  | Thailand |
| *Ophiocordyceps camponoti-nidulantis* | NIDUL2 | *Camponotus nidulans* | KX713640 | KX713611 | KX713669 | KX713717 |  | Brazil |
| *Ophiocordyceps camponoti-novogranadensis* | Mal63 | *Camponotus novogranadensis* | KX713648 | KX713603 |  |  |  | Brazil |
| *Ophiocordyceps camponoti-novogranadensis* | Mal4 | *Camponotus novogranadensis* | KX713649 | KX713602 |  |  |  | Brazil |
| *Ophiocordyceps camponoti-renggeri* | RENG2 | *Camponotus renggeri* | KX713632 |  | KX713672 |  |  | Brazil |
| *Ophiocordyceps camponoti-renggeri* | ORENG | *Camponotus renggeri* | KX713634 | KX713617 | KX713671 |  |  | Brazil |
| *Ophiocordyceps camponoti-rufipedis* | G177 | *Camponotus rufipes* | KX713657 | KX713596 | KX713680 |  |  | Brazil |
| *Ophiocordyceps camponoti-rufipedis* | G108 | *Camponotus rufipes* | KX713659 | KX713594 | KX713679 | KX713704 |  | Brazil |
| *Ophiocordyceps camponoti-saundersi* | C40 | *Camponotus saundersi* | KJ201519 |  | JN819012 |  |  | Thailand |
| *Ophiocordyceps camponoti-saundersi* | Co19 | *Camponotus saundersi* |  |  | JN819018 |  |  | Thailand |
| *Ophiocordyceps citrina* | TNS F18537 | Hemiptera |  | KJ878903 | KJ878983 |  | KJ878954 | Japan |
| *Ophiocordyceps clavata* | CEM 1762 | Coleoptera | KJ878916 | KJ878882 | KJ878963 | KJ878996 |  | China |
| *Ophiocordyceps cochlidiicola* | HMAS_199612 | Lepidoptera | KJ878917 | KJ878884 | KJ878965 | KJ878998 |  | China |
| *Ophiocordyceps contiispora* | YFCC 9025 | *Camponotus* sp. | ON555829 | ON555911 | ON567749 | ON568139 | ON568122 | China |
| *Ophiocordyceps contiispora* | YHH 20145 | *Camponotus* sp. | ON555830 |  | ON567750 | ON568140 | ON568123 | China |
| *Ophiocordyceps contiispora* | YFCC 9026 | *Camponotus* sp. | ON555831 | ON555912 | ON567751 | ON568141 | ON568124 | China |
| ***Ophiocordyceps contiispora*** | **YFCC 9027** | ***Camponotus* sp.** | **ON555832** | **ON555913** | **ON567752** | **ON568142** | **ON568125** | **China** |
| *Ophiocordyceps curculionum* | OSC 151910 | Coleoptera | KJ878918 | KJ878885 |  | KJ878999 |  | Guyana |
| *Ophiocordyceps daceti* | MF01 | *Daceton armigerum* |  | KX713604 | KX713667 |  |  | Brazil |
| *Ophiocordyceps dipterigena* | OSC 151911 | Diptera | KJ878919 | KJ878886 | KJ878966 | KJ879000 |  | USA |
| *Ophiocordyceps dipterigena* | OSC 151912 | Diptera | KJ878920 | KJ878887 | KJ878967 | KJ879001 |  | USA |
| *Ophiocordyceps flabellata* | YFCC 8795 | Hymenoptera (*Camponotus* sp.) | OL310721 | OL310724 | OL322688 | OL322687 | OL322695 | China |
| ***Ophiocordyceps flabellata*** | **YFCC 8796** | **Hymenoptera (*Camponotus* sp.)** | **OL310722** | **OL310723** | **OL322692** | **OL322689** | **OL322696** | **China** |
| *Ophiocordyceps formicarum* | TNSF 18565 | Hymenoptera | KJ878921 | KJ878888 | KJ878968 | KJ879002 | KJ878946 | Japan |
| *Ophiocordyceps formosana* | TNMF 13893 | Coleoptera | KJ878908 |  | KJ878956 | KJ878988 | KJ878943 | Taiwan, China |
| *Ophiocordyceps forquignonii* | OSC 151902 | Diptera | KJ878912 | KJ878876 |  | KJ878991 | KJ878945 | France |
| *Ophiocordyceps forquignonii* | OSC 151908 | Diptera | KJ878922 | KJ878889 |  | KJ879003 | KJ878947 | France |
| ***Ophiocordyceps fusiformispora*** | **YFCC 9014** | ***Polyrhachis* sp.** | **OR676962** | **OR685708** | **0R946326** |  | **PP091961** | **China** |
| ***Ophiocordyceps fusiformispora*** | **YFCC 9015** | ***Polyrhachis* sp.** | **OR676963** | **OR685709** | **0R946327** |  | **PP091962** | **China** |
| ***Ophiocordyceps fusiformispora*** | **YFCC 9045** | ***Polyrhachis* sp.** | **OR676964** | **OR685710** | **0R946328** |  | **PP091963** | **China** |
| ***Ophiocordyceps fusiformispora*** | **YFCC 9046** | ***Polyrhachis* sp.** | **OR676965** | **OR685711** | **0R946329** |  | **PP091964** | **China** |
| ***Ophiocordyceps fusiformispora*** | **YFCC 9047** | ***Polyrhachis* sp.** | **OR676966** | **OR685712** | **0R946330** |  | **PP091965** | **China** |
| *Ophiocordyceps ghanensis* | Gh41 | *Polyrhachis* sp. | KX713656 |  | KX713668 | KX713706 |  | Ghana |
| *Ophiocordyceps halabalaensis* | MY1308 | *Camponotus gigus* | KM655825 |  | GU797109 |  |  | Thailand |
| *Ophiocordyceps halabalaensis* | MY5151 | *Camponotus gigas* | KM655826 |  | GU797110 |  |  | Thailand |
| *Ophiocordyceps irangiensis* | OSC 128577 | Hymenoptera | DQ522546 | DQ518760 | DQ522329 | DQ522374 | DQ522427 |  |
| *Ophiocordyceps irangiensis* | OSC 128578 | Hymenoptera | DQ522556 | DQ518770 | DQ522345 | DQ522391 | DQ522445 |  |
| *Ophiocordyceps irangiensis* | OSC 128579 | Hymenoptera | EF469123 | EF469076 | EF469060 | EF469089 | EF469107 |  |
| *Ophiocordyceps kimflemingiae* | SC30 | *Camponotus castaneus*/*americanus* | KX713629 | KX713622 | KX713699 | KX713727 |  | USA |
| *Ophiocordyceps kimflemingiae* | SC09B | *Camponotus castaneus*/*americanus* | KX713631 | KX713620 | KX713698 | KX713724 |  | USA |
| *Ophiocordyceps kniphofioides* | HUA 186148 | Hymenoptera | KC610790 | KF658679 | KC610739 | KF658667 | KC610717 | Colombia |
| *Ophiocordyceps konnoana* | EFCC 7295 | Coleoptera | EF468958 |  |  | EF468862 | EF468915 | Korea |
| *Ophiocordyceps konnoana* | EFCC 7315 | Coleoptera | EF468959 |  | EF468753 | EF468861 | EF468916 | Korea |
| *Ophiocordyceps lilacina* | YHH 2210001 | *Polyrhachis* sp. | OP782343 |  | OP796856 | OP796861 |  | China |
| *Ophiocordyceps lilacina* | YHH 2210002 | *Polyrhachis sp.* | OP782344 |  | OP796857 | OP796862 |  | China |
| *Ophiocordyceps lloydii* | OSC 151913 | Hymenoptera | KJ878924 | KJ878891 | KJ878970 | KJ879004 | KJ878948 | Ecuador |
| *Ophiocordyceps longissima* | TNSF 18448 | Hemiptera | KJ878925 | KJ878892 | KJ878971 | KJ879005 |  | Japan |
| *Ophiocordyceps longissima* | HMAS_199600 | Hemiptera | KJ878926 |  | KJ878972 | KJ879006 | KJ878949 | China |
| *Ophiocordyceps melolonthae* | OSC 110993 | Coleoptera | DQ522548 | DQ518762 | DQ522331 | DQ522376 |  |  |
| *Ophiocordyceps melolonthae* | Ophgrc679 | Coleoptera |  | KC610768 | KC610744 | KF658666 |  | Colombia |
| *Ophiocordyceps monacidis* | MF74C | *Dolichoderus bispinosus* | KX713646 | KX713606 |  |  |  | Bazil |
| *Ophiocordyceps monacidis* | MF74 | *Dolichoderus bispinosus* | KX713647 | KX713605 |  | KX713712 |  | Bazil |
| *Ophiocordyceps myrmecophila* | CEM 1710 | Hymenoptera | KJ878928 | KJ878894 | KJ878974 | KJ879008 |  | China |
| *Ophiocordyceps naomipierceae* | DAWKSANT | *Polyrhachis* cf. *robsonii* | KX713664 | KX713589 |  | KX713701 |  | Australia |
| *Ophiocordyceps neovolkiana* | OSC 151903 | Coleoptera | KJ878930 | KJ878896 | KJ878976 | KJ879010 |  | Japan |
| *Ophiocordyceps nigrella* | EFCC 9247 |  | EF468963 | EF468818 | EF468758 | EF468866 | EF468920 | Korea |
| *Ophiocordyceps nooreniae* | BRIP 55363 | *Chariomyrma* cf. *hookeri* and *Polyrhachis* *lydiae* | NG065096 | NG059720 | KX673812 |  | KX673809 | Australia |
| *Ophiocordyceps nooreniae* | BRIP 64868 | *Polyrhachis* cf. *hookeri* and *Polyrhachis lydiae* | KX961142 |  | KX961143 |  |  | Australia |
| *Ophiocordyceps nutans* | OSC 110994 | Hemiptera | DQ522549 | DQ518763 | DQ522333 | DQ522378 |  |  |
| *Ophiocordyceps nuozhaduensis* | YHH 20168 | *Camponotus* sp. | ON555849 | ON555927 | ON567769 | ON568683 |  | China |
| *Ophiocordyceps nuozhaduensis* | YHH 20169 | *Camponotus* sp. | ON555850 | ON555928 | ON567770 | ON568684 |  | China |
| *Ophiocordyceps odonatae* | TNSF 18563 | Odonata |  | KJ878877 |  | KJ878992 |  | Japan |
| *Ophiocordyceps odonatae* | TNS F27117 | Odonata |  | KJ878878 |  |  |  | Japan |
| *Ophiocordyceps oecophyllae* | OECO1 | *Oecophyllas maragdina* | KX713635 |  |  |  |  | Australia |
| *Ophiocordyceps ootakii* | J14 | *Polyrhachis moesta* | KX713651 |  | KX713682 | KX713709 |  | Japan |
| *Ophiocordyceps ootakii* | J13 | *Polyrhachis moesta* | KX713652 | KX713600 | KX713681 | KX713708 |  | Japan |
| *Ophiocordyceps ponerinarum* | HUA 186140 | *Paraponera clavata* | KC610789 | KC610767 | KC610740 | KF658668 |  | Brazil |
| *Ophiocordyceps polyrhachis-furcata* | P39 | *Polyrhachis furcata* | KJ201504 |  | JN819003 |  |  | Thailand |
| *Ophiocordyceps polyrhachis-furcata* | P51 | *Polyrhachis furcata* | KJ201505 |  | JN819000 |  |  | Thailand |
| *Ophiocordyceps pulvinata* | TNS-F-30044 | *Camponotus obscuripes* | GU904208 |  | GU904209 | GU904210 |  | Japan |
| *Ophiocordyceps purpureostromata* | TNS F18430 | Coleoptera | KJ878931 | KJ878897 | KJ878977 | KJ879011 |  | Japan |
| *Ophiocordyceps rami* | MY6736 | *Camponotus* sp. | KM655823 |  | KJ201532 |  |  | Thailand |
| *Ophiocordyceps rami* | MY6738 | *Camponotus* sp. | KM655824 |  | KJ201534 |  |  | Thailand |
| *Ophiocordyceps ravenelii* | OSC 151914 | Coleoptera | KJ878932 |  | KJ878978 | KJ879012 | KJ878950 | USA |
| *Ophiocordyceps rhizoidea* | NHJ 12529 | Coleoptera | EF468969 | EF468824 | EF468765 | EF468872 | EF468922 |  |
| *Ophiocordyceps rhizoidea* | NHJ 12522 | Coleoptera | EF468970 | EF468825 | EF468764 | EF468873 | EF468923 |  |
| *Ophiocordyceps satoi* | J19 | *Polyrhachis lamellidens* | KX713650 | KX713601 | KX713684 | KX713710 |  | Japan |
| *Ophiocordyceps satoi* | J7 | *Polyrhachis lamellidens* | KX713653 | KX713599 | KX713683 | KX713711 |  | Japan |
| ***Ophiocordyceps satoi*** | **YFCC 8807** | ***Polyrhachis* sp.** | **OP782340** | **OP782345** | **OP796853** | **OP796858** | **OP796863** | **China** |
| *Ophiocordyceps septa* | Pur1 | *Camponotus* sp. |  |  | KJ201528 |  |  | Thailand |
| *Ophiocordyceps septa* | Pur2 | *Camponotus* sp. |  |  | KJ201529 |  |  | Thailand |
| *Ophiocordyceps sinensis* | EFCC 7287 | Lepidoptera | EF468971 | EF468827 | EF468767 | EF468874 | EF468924 |  |
| *Ophiocordyceps sobolifera* | KEW 78842 | Hemiptera | EF468972 | EF468828 |  | EF468875 | EF468925 |  |
| *Ophiocordyceps sphecocephala* | OSC 110998 | Hymenoptera | DQ522551 | DQ518765 | DQ522336 | DQ522381 | DQ522432 |  |
| *Ophiocordyceps stylophora* | OSC 111000 | Coleoptera | DQ522552 | DQ518766 | DQ522337 | DQ522382 | DQ522433 |  |
| *Ophiocordyceps stylophora* | OSC 110999 | Coleoptera | EF468982 | EF468837 | EF468777 | EF468882 | EF468931 |  |
| *Ophiocordyceps subtiliphialida* | YFCC 8815 | *Camponotus* sp. | ON555833 | ON555914 | ON567753 | ON568673 | ON568126 | China |
| *Ophiocordyceps subtiliphialida* | YFCC 8814 | *Camponotus* sp. | ON555834 | ON555915 | ON567754 | ON568674 | ON568127 | China |
| ***Ophiocordyceps subtiliphialida*** | **YFCC 8816** | ***Camponotus* sp.** | **ON555835** | **ON555916** | **ON567755** | **ON568675** | **ON568128** | **China** |
| *Ophiocordyceps subtiliphialida* | YFCC 8817 | *Camponotus* sp. | ON555836 | ON555917 | ON567756 | ON568676 | ON568129 | China |
| *Ophiocordyceps tianshanensis* | MFLU 19-1207 | *Camponotus japonicu*s | MN025409 | MN025407 | MK992784 |  |  | China |
| *Ophiocordyceps tianshanensis* | MFLU 19-1208 | *Camponotus japonicus* | MN025410 | MN025408 | MK992785 |  |  | China |
| *Ophiocordyceps tricentri* | CEM 160 | Hemiptera | AB027330 | AB027376 |  |  |  |  |
| *Ophiocordyceps unilateralis* | VIC 44303 | *Camponotus sericeiventris* | KX713628 | KX713626 | KX713675 | KX713730 |  | Brazil |
| *Ophiocordyceps unilateralis* | VIC 44354 | *Camponotus sericeiventris* | KX713627 |  | KX713676 | KX713731 |  | Brazil |
| *Ophiocordyceps yakusimensis* | HMAS_199604 | Hemiptera | KJ878938 | KJ878902 |  | KJ879018 | KJ878953 | China |
| *Tolypocladium inflatum* | OSC 71235 | Coleoptera | EF469124 | EF469077 | EF469061 | EF469090 | EF469108 |  |
| *Tolypocladium ophioglossoides* | CBS 100239 | *Elaphomyces* sp. | KJ878910 | KJ878874 | KJ878958 | KJ878990 | KJ878944 |  |

Table S2. Genomic assembly of seven species in *Ophiocordyceps unilateralis* complex.

| **Item** | **Value** | | | | | | |
| --- | --- | --- | --- | --- | --- | --- | --- |
|  | *O. contiispora* | *O. subtiliphialida* | *O. satoi* | *O. flabellata* | *O. acroasca* | *O. camponoti-leonardi* | *O. fusiformispora* |
| Genome Size (Mb) | 34.44 | 27.80 | 75.05 | 43.70 | 30.87 | 48.26 | 51.00 |
| GC content (%) | 46.67 | 53.22 | 44.05 | 44.77 | 50.11 | 45.88 | 41.81 |
| Total genes number | 6,756 | 6,700 | 6,760 | 6,836 | 6,974 | 6,879 | 6,706 |
| Average CDS length (bp) | 1,496.51 | 1,518.16 | 1,509.93 | 1,530.78 | 1,528.94 | 1,533.49 | 1,510.53 |
| Average gene length (bp) | 1,708.67 | 1,763.20 | 1,740.19 | 1,784.31 | 1,752.14 | 1770.39 | 1,740.39 |
| Average exon per gene | 2.55 | 2.59 | 2.58 | 2.60 | 2.64 | 2.60 | 2.60 |
| Average exon length (bp) | 589.57 | 587.87 | 594.47 | 598.79 | 580.14 | 593.97 | 582.52 |
| Average intron length (bp) | 130.56 | 150.16 | 129.37 | 144.11 | 133.08 | 139.79 | 139.04 |
| N50 (bp) | 68,012 | 68,181 | 11,763 | 42,780 | 79,664 | 124,761 | 12,740 |
| N90 (bp) | 8,398 | 13,673 | 176 | 8,216 | 22,435 | 31,027 | 149 |

Table S3. Functional annotation of seven species of *Ophiocordyceps unilateralis* sensu lato.

| **Item** | **Count/Percentage (%)** | | | | | | |
| --- | --- | --- | --- | --- | --- | --- | --- |
|  | *O. contiispora* | *O. subtiliphialida* | *O. satoi* | *O. flabellata* | *O. acroasca* | *O. camponoti-leonardi* | *O. fusiformispora* |
| KEGG | 2,130/31.53 | 2,116/31.58 | 2,104/31.12 | 2,126/31.10 | 2,153/30.87 | 2,135/31.04 | 2,131/31.78 |
| NR | 6,737/99.72 | 6,687/99.81 | 6,727/99.51 | 6,812/99.65 | 6,946/99.60 | 6,868/99.84 | 6,690/99.76 |
| GO | 5,065/74.97 | 5,041/75.24 | 5,123/75.78 | 5,204/76.13 | 5,208/74.68 | 5,258/76.44 | 5,112/76.23 |
| KOG | 10/0.15 | 10/0.15 | 9/0.13 | 5/0.07 | 12/0.17 | 3/0.04 | 5/0.07 |
| Pfam | 5,136/76.02 | 5,122/76.45 | 5,126/75.83 | 5,196/76.01 | 5,242/75.16 | 5,257/76.42 | 5,099/76.04 |
| Interpro | 6,571/97.26 | 6,551/97.78 | 6,540/96.75 | 6,660/97.43 | 6,800/97.51 | 6,707/97.50 | 6,546/97.61 |

Table S4. Genomes basic characteristics of twelve species in *Ophiocordyceps unilateralis* complex.

| **Item** | **Contig** | **Genome Size (Mb)** | **GC content (%)** | **N50 (bp)** | **Total genes number** |
| --- | --- | --- | --- | --- | --- |
| *O. contiispora* | 6,111 | 34.44 | 46.67 | 68,012 | 6,756 |
| *O. subtiliphialida* | 3,780 | 27.80 | 53.22 | 68,181 | 6,700 |
| *O. camponoti-rufipedis* | 2,257 | 21.90 | 56.10 | 23,042 | 7,618 |
| *O. camponoti-floridani* | 13 | 30.47 | 48.41 | 3,776,764 | 7,455 |
| *O. satoi* | 76,509 | 75.05 | 44.05 | 11,763 | 6,760 |
| *O. polyrhachis-furcata* | 3,555 | 43.25 | 43.30 | 27,154 | 10,146 |
| *O. flabellata* | 9,875 | 43.70 | 44.77 | 42,780 | 6,836 |
| *O. camponoti-saundersi* | 2,184 | 49.27 | 40.00 | 60,659 | 6,946 |
| *O. acroasca* | 1,053 | 30.87 | 50.11 | 79,664 | 6,974 |
| *O. camponoti-leonardi* | 1,842 | 48.26 | 45.88 | 124,761 | 6,879 |
| *O. fusiformispora* | 65,838 | 51.00 | 41.81 | 12,740 | 6,706 |
| *O. unilateralis* | 3,969 | 23.91 | 55.50 | 20,827 | 8,577 |

Table S5. The biosynthesis gene cluster of putative secondary metabolites in twelve species of *Ophiocordyceps unilateralis* sensu lato.

| Species | BGCs | Location | From | To | Domain | Type | Most similar known cluster (%) |
| --- | --- | --- | --- | --- | --- | --- | --- |
| *O. polyrhachis-furcata* | Region 1.4 | Scaffold1.g46 | 235055 | 238650 | A-P-Te | NRPS | Unknown |
|  | Region 4.1 | Scaffold4.g159 | 629293 | 633394 | A | NRPS | Pyranonigrin E (100%) |
|  | Region 6.1 | Scaffold6.g50 | 185783 | 198636 | A-C-A-P-C-A-P-C-A-P-C | NRPS | Unknown |
|  | Region 10.1 | Scaffold10.g118 | 570459 | 574366 | A-Te | NRPS | Unknown |
|  | Region 17.1 | Scaffold17.g3 | 44110 | 54361 | A-C-P | NRPS | Unknown |
|  | Region 41.2 | Scaffold41.g214 | 2604162 | 2606939 | A-P-Te | NRPS | Unknown |
|  | Region 61.3 | Scaffold61.g204 | 1014825 | 1017357 | C-A-P | NRPS | Unknown |
|  | Region 67.1 | Scaffold67.g148 | 22450 | 30545 | A-P | NRPS | Unknown |
|  | Region 61.4 | Scaffold61.g406 | 1808202 | 1813498 | CaiC-P-T | NRPS,Other | Unknown |
|  | Region 2.1 | Scaffold2.g87 | 429048 | 440950 | KS-AT-MT-KR-P-C-A-P-Te | PKS,NRPS | NG-391 (100%) |
|  | Region 1.2 | Scaffold1.g21 | 141329 | 148450 | KS-AT-DH-ER-KR-ACP | HR-PKS | Unknown |
|  | Region 1.3 | Scaffold1.g24 | 157353 | 165492 | KS-AT-DH-MT-ER-KR-ACP | HR-PKS | Unknown |
|  | Region 2.2 | Scaffold2.g138 | 636761 | 645359 | KS-AT-DH-MT-ER-KR-ACP | HR-PKS | Unknown |
|  | Region 4.2 | Scaffold4.g263 | 1041201 | 1050852 | PT-KS-AT-DH-MT-ER-KR-ACP | HR-PKS | Unknown |
|  | Region 6.2 | Scaffold6.g124 | 535987 | 543842 | KS-AT-DH-ER-KR-ACP | HR-PKS | 4-epi-15-epi-brefeldin (20%) |
|  | Region 61.5 | Scaffold61.g513 | 2205534 | 2215386 | KS-AT-DH-MT-ER-KR-ACP | HR-PKS | Unknown |
|  | Region 61.6 | Scaffold61.g755 | 3455680 | 3463508 | KS-AT-DH-ER-KR-ACP | HR-PKS | Unknown |
|  | Region 1.1 | Scaffold1.g19 | 126910 | 134895 | SAT-KS-AT-PT-ACP-ACP-ACP-Te | NR-PKS | Viriditoxin (22%) |
|  | Region 5.2 | Scaffold5.g94 | 364621 | 371124 | SAT-KS-AT-PT-ACP-HTH-MT | NR-PKS | Chrodrimanin B (30%) |
|  | Region 25.1 | Scaffold25.g6 | 57933 | 64443 | SAT-KS-AT-PT-ACP-ACP-Te | NR-PKS | Unknown |
|  | Region 41.1 | Scaffold41.g98 | 689179 | 695219 | SAT-KS-AT-PT-ACP-Te | NR-PKS | Aflatoxin (8%) |
|  | Region 61.1 | Scaffold61.g86 | 393622 | 399528 | SAT-KS-AT-PT-ACP-ACP | NR-PKS | Unknown |
|  | Region 61.2 | Scaffold61.g200 | 998398 | 1004149 | SAT-KS-AT-PT | NR-PKS | Cichorine (100%) |
|  | Region 5.2 | Scaffold5 | 345356 | 386211 |  | PKS | Chrodrimanin (30%) |
|  | Region 13.1 | Scaffold13 | 101881 | 122912 |  | Terpene | Squalestatin S1 (40%) |
|  | Region 16.1 | Scaffold16 | 821984 | 843450 |  | Indole | Unknown |
|  | Region 1.5 | Scaffold1.g133 | 545841 | 550908 | Te | Other | Unknown |
|  | Region 21.1 | Scaffold21 | 67687 | 103508 |  | Terpene | Terpendole E (100%) |
| *O. contiispora* | Region 3.1 | NODE3.g28 | 86362 | 96548 | A-P | NRPS | Unknown |
|  | Region 9.1 | NODE9.g57 | 66915 | 74465 | A-P | NRPS | Unknown |
|  | Region 20.1 | NODE20.g33 | 146243 | 158959 | A-P-A-P-C-A-P-C | NRPS | Unknown |
|  | Region 23.1 | NODE23.g44 | 147590 | 162255 | P-A-C-P-C-P-C | NRPS | Unknown |
|  | Region 30.1 | NODE30.g23 | 54915 | 100395 | A-MT-Te | NRPS | Unknown |
|  | Region 34.1 | NODE34.g27 | 99201 | 102674 | A-Te | NRPS | Unknown |
|  | Region 48.1 | NODE48.g14 | 58078 | 61654 | A-P-Lys2b | NRPS,Other | Unknown |
|  | Region 28.1 | NODE28.g24 | 132861 | 144883 | KS-AT-DH-MT-KR-P-C-A-P-Te | PKS,NRPS | NG-391 (83%) |
|  | Region 14.1 | NODE14.g29 | 85335 | 92885 | A-P-KS-AT-KR-ACP-Te | PKS,NRPS | Swainsonine (28%) |
|  | Region 22.2 | NODE22.g21 | 74484 | 81610 | KS-AT-ER-KR-ACP | HR-PKS | Unknown |
|  | Region 22.3 | NODE22.g24 | 89530 | 97727 | KS-AT-MT-ER-KR-ACP | HR-PKS | Unknown |
|  | Region 80.1 | NODE80.g6 | 26384 | 34509 | KS-AT-DH-MT-ER-KR-ACP | HR-PKS | Phenalamide (40%) |
|  | Region 25.2 | NODE25.g16 | 43553 | 52836 | KS-AT-DH-KR-ACP-4CL | PR-NRPS | Pyripyropene A (68%) |
|  | Region 3.4 | NODE3.g56 | 212980 | 219884 | KS-AT-MT-ER | PR-NRPS | Pyripyropene A (33%) |
|  | Region 5.1 | NODE5.g19 | 61407 | 66671 | KS-AT-DH-MT-ER-ACP-ACP | PR-NRPS | Gephyronic acid (19%) |
|  | Region 3.3 | NODE3.g54 | 200831 | 207308 | SAT-KS-AT-PT-ACP-HTH-MT | NR-PKS | Higginsianin B (80%) |
|  | Region 21.1 | NODE21.g24 | 97176 | 98449 | AT | NR-PKS | Formicamycins A-M (4%) |
|  | Region 22.1 | NODE22.g19 | 63259 | 70605 | SAT-KS-AT-PT-ACP-ACP-Te | NR-PKS | Phenalamide (16%) |
|  | Region 37.1 | NODE37.g27 | 99689 | 106277 | SAT-KS-AT-PT-ACP-ACP-Te | NR-PKS | Unknown |
|  | Region 103.1 | NODE103 | 1 | 34676 |  | T3PKS | Unknown |
|  | Region3.1 | NODE3 | 66362 | 12939 |  | Terpene | Clavaric acid (100%) |
|  | Region 17.1 | NODE17 | 146144 | 167029 |  | Terpene | Unknown |
|  | Region 25.1 | NODE25 | 1 | 16660 |  | Terpene | Squalestatin S1 (40%) |
|  | Region 46.1 | NODE46 | 43077 | 64168 |  | Terpene | Terpendole (57%) |
|  | Region 86.1 | NODE86 | 63594 | 84208 |  | Terpene | Unknown |
|  | Region 352.1 | NODE352 | 1 | 17549 |  | Terpene | Unknown |
|  | Region 2.1 | NODE2.g98 | 349930 | 353002 | AFD-P-Te | Other | Unknown |
| *O. flabellata* | Region 9.1 | NODE9.g5 | 17042 | 29620 | A-P-A-P-A-C-A-P-C | NRPS | Unknown |
|  | Region 36.1 | NODE36.g1 | 659 | 10288 | A-A-P | NRPS | Unknown |
|  | Region 64.2 | NODE64.g10 | 39768 | 42515 | C-A-P | NRPS | Unknown |
|  | Region 89.1 | NODE89.g13 | 34767 | 45498 | A-C-P | NRPS | Cichorine (100%) |
|  | Region 95.1 | NODE95.g3 | 13663 | 16533 | A-P-Te | NRPS | Unknown |
|  | Region 262.1 | NODE262.g3 | 8050 | 11565 | A-Te | NRPS | Unknown |
|  | Region 378.1 | NODE378.g2 | 3989 | 18358 | A-A-P-C-P-C | NRPS | Unknown |
|  | Region 506.1 | NODE506.g3 | 8452 | 11592 | A-P-Te | NRPS | Unknown |
|  | Region 13.2 | NODE13.g12 | 50138 | 55040 | CaiC-P-T | NRPS,Other | Unknown |
|  | Region 239.1 | NODE239.g4 | 19781 | 23371 | A-P-Lys2b | NRPS,Other | Unknown |
|  | Region 72.1 | NODE72.g10 | 22113 | 33566 | KS-AT-DH-MT-KR-P-C-A-P-Te | PKS,NRPS | NG-391 (100%) |
|  | Region 143.1 | NODE143.g9 | 46991 | 55689 | A-P-KS-AT-KR-ACP-Te | PKS,NRPS | Swainsonine (28%) |
|  | Region 3.2 | NODE3.g9 | 35755 | 44365 | KS-AT-MT-ER-KR-ACP | HR-PKS | Pyripyropene A (33%) |
|  | Region 9.2 | NODE9.g29 | 129292 | 136612 | KS-AT-DH-ER-KR-ACP | HR-PKS | Unknown |
|  | Region 13.1 | NODE13.g11 | 42251 | 49829 | KS-AT-DH-MT-ER-KR-ACP | HR-PKS | 1-heptadecene (100%) |
|  | Region 59.1 | NODE59.g17 | 71637 | 79904 | KS-AT-MT-ER-KR-ACP | HR-PKS | Unknown |
|  | Region 82.1 | NODE82.g3 | 9339 | 16541 | KS-AT-ER-KR-ACP | HR-PKS | 4-epi-15-epi-brefeldin (20%) |
|  | Region 140.1 | NODE140.g5 | 28365 | 34771 | AT-DH-MT-ER-KR-ACP | HR-PKS | Viriditoxin (22%) |
|  | Region 140.2 | NODE140.g8 | 45178 | 52301 | KS-AT-DH-MT-ER-KR-ACP | HR-PKS | Unknown |
|  | Region 179.1 | NODE179.g3 | 14582 | 22362 | KS-AT-DH-ER-KR-ACP-Te | HR-PKS | Unknown |
|  | Region 257.1 | NODE257.g8 | 32543 | 40635 | KS-AT-DH-MT-ER-KR-ACP | HR-PKS | Epothilone B (13%) |
|  | Region 111.1 | NODE111.g2 | 10462 | 19307 | KS-AT-DH-MT-ER-ACP-ACP | PR-PKS | Unknown |
|  | Region 3.1 | NODE3.g7 | 24630 | 31172 | SAT-KS-AT-PT-ACP-HTH-MT | NR-PKS | Pyripyropene A (33%) |
|  | Region 64.1 | NODE64.g6 | 21707 | 28194 | KS-AT-PT-ACP-Te | NR-PKS | Cichorine (100%) |
|  | Region 140.3 | NODE140.g10 | 56320 | 63814 | SAT-KS-AT-PT-ACP-ACP-ACP-Te | NR-PKS | Unknown |
|  | Region 600.1 | NODE600.g3 | 5156 | 11730 | SAT-KS-AT-PT-ACP-ACP-Te | NR-PKS | Unknown |
|  | Region 182.1 | NODE182 | 14496 | 55587 |  | T3PKS | Unknown |
|  | Region 32.1 | NODE32 | 29321 | 50523 |  | Indole | Unknown |
|  | Region 36.2 | NODE36 | 59783 | 81420 |  | Terpene | Clavaric acid (100%) |
|  | Region 431.1 | NODE431 | 1 | 32563 |  | Terpene | Terpendole E (100%) |
|  | Region 446.1 | NODE446 | 12930 | 30651 |  | Terpene | Unknown |
|  | Region 31.1 | NODE31.g27 | 89494 | 92334 | AFD-P-Te | Other | Unknown |
| *O. acroasca* | Region 1.2 | NODE1.g20 | 78431 | 88609 | A-P | NRPS | Unknown |
|  | Region 2.1 | NODE2.g63 | 263898 | 277873 | P-A-C-P-P-C | NRPS | Unknown |
|  | Region 22.2 | NODE22.g15 | 72489 | 83204 | A-P | NRPS | Unknown |
|  | Region 3.1 | NODE3.g35 | 134272 | 137611 | A-P-Te | NRPS | Unknown |
|  | Region 49.1 | NODE49.g11 | 59570 | 64490 | P-C-A-P-C | NRPS | Unknown |
|  | Region 49.2 | NODE49.g17 | 79851 | 91622 | A-P-A-P-A-C-A-P-C | NRPS | Unknown |
|  | Region 104.1 | NODE104.g23 | 74549 | 78058 | A-Te | NRPS | Unknown |
|  | Region 145.2 | NODE145.g4 | 14281 | 22591 | CaiC-P-T | NRPS,Other | Ajudazol A (46%) |
|  | Region 265.1 | NODE265.g7 | 15477 | 23610 | C-A-MT-Te-HutI | NRPS,Other | Unknown |
|  | Region 388.1 | NODE388.g4 | 11222 | 14800 | A-P-Lys2b | NRPS,Other | Unknown |
|  | Region 55.1 | NODE55.g1 | 7912 | 15381 | A-P-KS-AT-ER-ACP-Te | PKS,NRPS | Swainsonina (28%) |
|  | Region 1.4 | NODE1.g51 | 214650 | 223101 | KS-AT-DH-MT-ER-KR-ACP | HR-PKS | Leucinostatin A/B (10%) |
|  | Region 44.1 | NODE44.g13 | 54062 | 62149 | KS-AT-DH-MT-ER-KR-ACP | HR-PKS | Pellasoren (33%) |
|  | Region 58.2 | NODE58.g16 | 87963 | 95078 | KS-AT-ER-KR-ACP | HR-PKS | Unknown |
|  | Region 58.3 | NODE58.g19 | 104910 | 113194 | KS-AT-DH-MT-ER-KR-ACP | HR-PKS | Unknown |
|  | Region 145.1 | NODE145.g3 | 8799 | 13507 | KS-AT-DH-MT-ER-KR-ACP | HR-PKS | Ajudazol A (46%) |
|  | Region 1.3 | NODE1.g49 | 201404 | 207879 | SAT-KS-AT-PT-ACP-HTH-MT | NR-PKS | Leucinostatin A/B (10%) |
|  | Region 15.1 | NODE15.g31 | 114571 | 120204 | SAT-KS-AT-PT-ACP | NR-PKS | Alternariol (100%) |
|  | Region 22.1 | NODE22.g13 | 55945 | 60846 | KS-AT-PT | NR-PKS | Cichorine (100%) |
|  | Region 58.1 | NODE58.g15 | 76299 | 83712 | SAT-KS-AT-PT-ACP-ACP-ACP-Te | NR-PKS | Naphthopyrone (100%) |
|  | Region 158.1 | NODE158.g3 | 4449 | 10918 | SAT-KS-AT-PT-ACP-ACP-Te | NR-PKS | Unknown |
|  | Region1.1 | NODE1 | 58431 | 116151 |  | Terpene | Clavaric acid (100%) |
|  | Region 75.1 | NODE75 | 54576 | 85504 |  | Terpene | Shearinine (18%) |
|  | Region 399.1 | NODE399 | 1 | 17210 |  | Terpene | Squalestatin S1 (40%) |
|  | Region 439.1 | NODE439 | 1927 | 15768 |  | Terpene | Unknown |
| *O. satoi* | Region 15.1 | NODE15 | 6459 | 27118 |  | NRPS | Unknown |
|  | Region 18.1 | NODE18.g12 | 31581 | 35511 | A-Te | NRPS | Leucinostatin A/B (10%) |
|  | Region 39.1 | NODE39.g7 | 22450 | 30545 | P-A-C-P-P-C | NRPS | Unknown |
|  | Region 84.1 | NODE84.g21 | 58794 | 69521 | A-C-P | NRPS | Unknown |
|  | Region 95.1 | NODE195.g11 | 29997 | 33413 | A-Te | NRPS | Unknown |
|  | Region 225.1 | NODE225.g4 | 17405 | 29932 | A-P-A-P-A-C-A-P-C | NRPS | Unknown |
|  | Region 393.1 | NODE393.g3 | 12724 | 22936 | P-A-C-A-P | NRPS | Unknown |
|  | Region 6.2 | NODE6.g37 | 135868 | 140988 | CaiC-P-T | NRPS,Other | Unknown |
|  | Region 8.2 | NODE8.g43 | 150501 | 161317 | AspB-A-MT-Te-HutI | NRPS,Other | Unknown |
|  | Region 14.1 | NODE14.g28 | 119263 | 126095 | A-P-Te-MT-CYP | NRPS,Other | Unknown |
|  | Region 72.1 | NODE72.g8 | 28846 | 32439 | A-P-Lys2b | NRPS,Other | Avilamycin A/C (5%) |
|  | Region 25.1 | NODE125.g6 | 11302 | 20832 | A-P-KS-AT-KR-ACP-Te | PKS,NRPS | Swainsonine (28%) |
|  | Region 66.1 | NODE66.g11 | 30947 | 50941 | KS-AT-DH-MT-KR-P-C-A-P-Te-MFS-Dehydrogenase-CYP-MT | PKS,NRPS,Other | Lucilactaene (100%) |
|  | Region 6.1 | NODE6.g36 | 127680 | 135645 | KS-AT-DH-MT-ER-KR-ACP | HR-PKS | Piericidin (25%) |
|  | Region 12.1 | NODE12.g26 | 103635 | 111740 | KS-AT-DH-MT-ER-KR-ACP | HR-PKS | Ajudazol A (30%) |
|  | Region 18.3 | NODE18.g25 | 73804 | 82177 | KS-AT-DH-MT-ER-KR-ACP | HR-PKS | Unknown |
|  | Region 54.1 | NODE54.g7 | 22450 | 30545 | KS-AT-DH-MT-ER-KR-ACP | HR-PKS | Pellasoren (33%) |
|  | Region 59.1 | NODE59.g11 | 49020 | 56371 | KS-AT-DH-MT-ER-KR-ACP | HR-PKS | Emericellamide A/B (40%) |
|  | Region 99.1 | NODE99.g1 | 298 | 6677 | SAT-KS-AT-PT-ACP-Te | NR-PKS | Viriditoxin (14%) |
|  | Region 111.1 | NODE111.g7 | 24121 | 30674 | SAT-KS-AT-PT-ACP-ACP-Te | NR-PKS | Unknown |
|  | Region 18.2 | NODE18.g23 | 62949 | 69489 | SAT-KS-AT-PT-ACP-HTH-MT | NR-PKS | Unknown |
|  | Region 127.1 | NODE127.g14 | 38070 | 45510 | SAT-KS-AT-PT-ACP-ACP-ACP-Te | NR-PKS | Naphthopyrone (100%) |
|  | Region 15.2 | NODE15 | 132069 | 164572 |  | T3PKS | Unknown |
|  | Region 3.1 | NODE3 | 161613 | 182626 |  | Terpene | Squalestatin S19 (40%) |
|  | Region 4.1 | NODE4 | 58090 | 92379 |  | Terpene | Terpendole E (100%) |
|  | Region 61.1 | NODE61 | 42801 | 64435 |  | Terpene | Unknown |
|  | Region 343.1 | NODE343 | 1 | 16194 |  | Indole | Unknown |
|  | Region 8.1 | NODE8.g20 | 74055 | 77333 | CaiC-P-Te | Other | Unknown |
| *O. subtiliphialida* | Region 2.1 | NODE2.g40 | 172018 | 182732 | A-P | NRPS | Unknown |
|  | Region 3.1 | NODE3.g12 | 32347 | 35865 | A-P-Te | NRPS | Unknown |
|  | Region 10.1 | NODE10.g13 | 42456 | 47387 | C-A-P | NRPS | Unknown |
|  | Region 10.2 | NODE10.g17 | 61866 | 74591 | A-C-A-P-C-A-P-C-A-P-C | NRPS | Unknown |
|  | Region 21.1 | NODE21.g16 | 69818 | 80936 | A-P-C-P-C-A-C-P-C-P-C | NRPS | Unknown |
|  | Region 27.2 | NODE27.g21 | 74271 | 84899 | A-C-A-P | NRPS | Unknown |
|  | Region 32.1 | NODE32 | 92095 | 137620 |  | NRPS | Unknown |
|  | Region 68.1 | NODE68.g12 | 31186 | 34424 | A-P-Te | NRPS | Unknown |
|  | Region 273.1 | NODE273.g3 | 12150 | 19628 | A-P-KS-AT-KR-ACP-Te | PKS,NRPS | Swainsonine (28%) |
|  | Region74.1 | NODE74.g6 | 27694 | 33030 | CaiC-P-T | NRPS,Other | Iasalocid (13%) |
|  | Region 286.1 | NODE286.g4 | 7820 | 11396 | A-P-Lys2b | NRPS,Other | Unknown |
|  | Region74.1 | NODE74.g7 | 33264 | 40352 | KS-AT-DH-MT-ER-KR-ACP | HR-PKS | Iasalocid (13%) |
|  | Region 184.1 | NODE184.g8 | 23002 | 31106 | KS-AT-DH-MT-ER-KR-ACP | HR-PKS | Pellasoren (41%) |
|  | Region 19.2 | NODE19.g13 | 49956 | 57148 | KS-AT-ER-KR-ACP | HR-PKS | Naphthopyrone (100%) |
|  | Region 19.3 | NODE19.g16 | 65743 | 74125 | KS-AT-DH-MT-ER-KR-ACP | HR-PKS | Naphthopyrone (100%) |
|  | Region 97.1 | NODE97.g6 | 42040 | 48554 | SAT-KS-AT-PT-ACP-ACP-Te | NR-PKS | Unknown |
|  | Region 14.1 | NODE14.g7 | 27284 | 32657 | SAT-KS-AT-PT-ACP | NR-PKS | Higginsianin B (80%) |
|  | Region 19.1 | NODE19.g11 | 37698 | 45186 | SAT-KS-AT-PT-ACP-ACP-ACP-Te | NR-PKS | Naphthopyrone (100%) |
|  | Region 40.1 | NODE40 | 38832 | 79767 |  | T3PKS | Unknown |
|  | Region27.1 | NODE27 | 49919 | 104899 |  | Terpene | Clavaric acid (100%) |
|  | Region 34.1 | NODE34 | 37673 | 58632 |  | Terpene | Terpendole E (57%) |
|  | Region 38.1 | NODE38 | 105104 | 124532 |  | Terpene | Unknown |
|  | Region 135.1 | NODE135 | 6794 | 27780 |  | Terpene | Unknown |
|  | Region 492.1 | NODE492 | 1 | 10487 |  | Terpene | Squalestatin S1 (40%) |
|  | Region 73.1 | NODE73 | 59593 | 80783 |  | Indole | Unknown |
| *O. camponoti-rufipedis* | Region 558.1 | NODE558.g1 | 2074 | 12939 | A-P | NRPS | Unknown |
|  | Region 890.1 | NODE890.g10 | 37172 | 41375 | A-P | NRPS | Unknown |
|  | Region 924.1 | NODE924.g6 | 14922 | 28451 | C-A-P-A-P-A-P-C | NRPS | Unknown |
|  | Region 1426.1 | NODE1426.g5 | 10701 | 21852 | P-A | NRPS | Unknown |
|  | Region 1997.1 | NODE1997.g10 | 25460 | 40141 | P-A-C-P-C-P-C | NRPS | Unknown |
|  | Region 2141.1 | NODE2141.g3 | 10700 | 21252 | A-C-P | NRPS | Unknown |
|  | Region 591.1 | NODE591.g4 | 11628 | 16430 | CaiC-P-T | NRPS,Other | Unknown |
|  | Region 935.1 | NODE935.g7 | 22302 | 25784 | A-Lys2b | NRPS,Other | Unknown |
|  | Region 1013.1 | NODE1013.g5 | 15169 | 19029 | C-A-Lys2b | NRPS,Other | Unknown |
|  | Region 1586.1 | NODE1586.g15 | 48705 | 51467 | CaiC-P-Te | NRPS,Other | Unknown |
|  | Region 1769.1 | NODE1769.g1 | 258 | 2834 | A-Lys2b | NRPS,Other | Unknown |
|  | Region 1794.1 | NODE1794.g4 | 4633 | 9228 | A-P-Lys2b | NRPS,Other | Unknown |
|  | Region 2030.1 | NODE2030.g10 | 29191 | 41488 | KS-AT-KR-P-C-A-P-Te | PKS,NRPS | NG-391 (66%) |
|  | Region 1375.1 | NODE1375.g11 | 35877 | 52818 | KS-AT-DH-MT-ER-KR-ACP | HR-PKS | Phenalamide (41%) |
|  | Region 2152.1 | NODE2152.g8 | 21137 | 28048 | KS-AT-ER-KR-ACP | HR-PKS | Viriditoxin (22%) |
|  | Region 668.1 | NODE668.g4 | 7602 | 14049 | SAT-KS-AT-PT-ACP-HTH-MT | NR-PKS | Higginsianin B (80%) |
|  | Region 1442.1 | NODE1442.g18 | 48422 | 54892 | SAT-KS-AT-PT-ACP-ACP-Te | NR-PKS | Secalonic (12%) |
|  | Region 2152.2 | NODE2152.g10 | 31519 | 38905 | SAT-KS-AT-PT-ACP-ACP-ACP-Te | NR-PKS | Unknown |
|  | Region 1542.1 | NODE11542 | 1952 | 42737 |  | T3PKS | Unknown |
|  | Region 113.1 | NODE113 | 23009 | 44766 |  | Terpene | Unknown |
|  | Region 358.1 | NODE358 | 9720 | 30403 |  | Terpene | Unknown |
|  | Region 1497.1 | NODE1497 | 5679 | 26266 |  | Terpene | Unknown |
|  | Region 1583.1 | NODE1583 | 1 | 15943 |  | Terpene | Squalestatin S1 (40%) |
|  | Region 1664.1 | NODE1446 | 23219 | 44343 |  | Terpene | Unknown |
|  | Region 1830.1 | NODE1830 | 10548 | 31882 |  | Terpene | Terpendole E (71%) |
|  | Region 1886.1 | NODE1886 | 15118 | 36131 |  | Terpene | Unknown |
| *O. camponoti-leonardi* | Region 1.1 | NODE1.g55 | 172710 | 182898 | A-A-P | NRPS | Unknown |
|  | Region 1.4 | NODE1.g146 | 531715 | 544308 | C-A-P-A-P-A-A-P-C | NRPS | Unknown |
|  | Region 4.2 | NODE4.g3 | 21071 | 22606 | P-Te | NRPS | Unknown |
|  | Region 17.1 | NODE17.g3 | 25186 | 28071 | A-P-Te | NRPS | Unknown |
|  | Region 34.1 | NODE34.g19 | 75588 | 89982 | P-A-C-P-C-P-C | NRPS | Unknown |
|  | Region 54.1 | NODE54.g4 | 43357 | 54088 | A-P | NRPS | Cichorine (100%) |
|  | Region 99.1 | NODE99.g14 | 58014 | 60775 | C-A | NRPS | Cichorine (1%) |
|  | Region 192.1 | NODE192.g8 | 20265 | 23783 | A-Te | NRPS | Unknown |
|  | Region 15.2 | NODE15.g34 | 117475 | 122723 | CaiC-P-T | NRPS,Other | Unknown |
|  | Region 21.1 | NODE21.g11 | 46645 | 50941 | A-P-NAD | NRPS,Other | Unknown |
|  | Region 127.1 | NODE127.g13 | 44885 | 52690 | A-P-KS-AT-KR-ACP-Te | PKS,NRPS | Swainsonine (28%) |
|  | Region 4.3 | NODE4.g69 | 253078 | 266176 | KS-AT-DH-MT-ER-KR-ACP-MPP | PKS,Other | Unknown |
|  | Region 211.1 | NODE 211.1 | 651 | 41607 |  | T3PKS | Unknown |
|  | Region 1.5 | NODE1.g1 | 920 | 3322 | ER-KR-ACP | HR-PKS | Unknown |
|  | Region 1.6 | NODE1.g88 | 299317 | 307914 | KS-AT-DH-MT-ER-KR-ACP | HR-PKS | Unknown |
|  | Region 15.1 | NODE15.g33 | 108891 | 117286 | KS-AT-MT-ER-KR-ACP | HR-PKS | Piericidin A1 (33%) |
|  | Region 40.1 | NODE40.g8 | 35245 | 43029 | KS-AT-DH-ER-KR-ACP-Te | HR-PKS | Unknown |
|  | Region 134.2 | NODE134.g14 | 53171 | 60309 | KS-AT-Cya-ER-KR-ACP | HR-PKS | Unknown |
|  | Region 134.3 | NODE134.g17 | 70962 | 79102 | KS-AT-MT-ER-KR-ACP | HR-PKS | Unknown |
|  | Region 160.1 | NODE160.g2 | 16818 | 25820 | KS-AT-DH-MT-ER-KR-ACP | HR-PKS | Unknown |
|  | Region 190.1 | NODE190.g8 | 30264 | 37596 | KS-AT-DH-ER-KR-ACP | HR-PKS | Unknown |
|  | Region 221.1 | NODE221.g1 | 306 | 7882 | KS-AT-DH-ER-KR-ACP | HR-PKS | Unknown |
|  | Region 1.3 | NODE1.g85 | 288370 | 294915 | SAT-KS-AT-PT-ACP-MTH-MT | NR-PKS | Leucinostatin A/B (10%) |
|  | Region 51.1 | NODE51.g14 | 51452 | 58025 | SAT-KS-AT-PT-ACP-ACP-Te | NR-PKS | Unknown |
|  | Region 94.1 | NODE94.g17 | 60171 | 66464 | SAT-KS-AT-PT-ACP-Te | NR-PKS | Viriditoxin (14%) |
|  | Region 99.2 | NODE99.g18 | 72306 | 78748 | SAT-KS-AT-PT-ACP-Aes | NR-PKS | Unknown |
|  | Region 134.1 | NODE134.g12 | 41553 | 49040 | SAT-KS-AT-PT-ACP-ACP-ACP-Te | NR-PKS | Viriditoxin (22%) |
|  | Region 1.2 | NODE1 | 233050 | 254987 |  | Terpene | Clavaric acid (100%) |
|  | Region 4.1 | NODE4.1 | 177839 | 198840 |  | Terpene | Squalestatin S1 (40%) |
|  | Region 35.1 | NODE35.1 | 30707 | 51603 |  | Terpene | Unknown |
|  | Region 39.1 | NODE39.1 | 82953 | 103921 |  | Terpene | Unknown |
|  | Region 61.1 | NODE61.1 | 137085 | 154338 |  | Terpene | Unknown |
|  | Region 151.1 | NODE151.1 | 18979 | 39941 |  | Terpene | Unknown |
|  | Region 152.1 | NODE152.1 | 18979 | 39941 |  | Terpene | Unknown |
|  | Region 5.1 | NODE 5.1 | 356947 | 369058 |  | Indole | Nodulisporic acid F (15%) |
|  | Region 19.1 | NODE 19.1 | 130065 | 168583 |  | Indole,Terpene | Terpendole E (100%) |
| *O. unilateralis* | Region 558.1 | NODE558.g1 | 156 | 14460 | P-A-C-P-P-C | NRPS | Unknown |
|  | Region 1729.1 | NODE1729.g2 | 2221 | 25680 | A-P-C-A-P-C | NRPS | Unknown |
|  | Region 1782.1 | NODE1782.g5 | 8933 | 19170 | A-P | NRPS | Unknown |
|  | Region 1850.1 | NODE1850.g9 | 29322 | 42890 | C-A-P-C-A-P-A-C-A-P-C | NRPS | Unknown |
|  | Region 2119.1 | NODE2119.g2 | 4703 | 8312 | C-A-P-Te | NRPS | Unknown |
|  | Region 2283.1 | NODE2283.g3 | 11995 | 15132 | A-P-Te | NRPS | Unknown |
|  | Region 502.1 | NODE502.g2 | 1352 | 4909 |  | NRPS-like | Unknown |
|  | Region 1775.1 | NODE1775 | 1 | 24264 |  | NRPS-like | Unknown |
|  | Region 1986.1 | NODE1986.g4 | 9025 | 12595 | A-P-Lys2b | NRPS,Other | Unknown |
|  | Region 2335.1 | NODE2335.g1 | 429 | 4145 | CaiC-P-T | NRPS,Other | Unknown |
|  | Region 913.1 | NODE913.g8 | 18257 | 30301 | KS-AT-DH-MT-KR-P-C-A-P-Te | PKS,NRPS | NG-391 (100%) |
|  | Region 757.1 | NODE757.g3 | 4939 | 12732 | KS-AT-DH-MT-ER-KR-ACP | HR-PKS | Beauveriolide B/C/D (33%) |
|  | Region 1784.1 | NODE1784.g9 | 29692 | 37876 | KS-AT-DH-MT-ER-KR-ACP | HR-PKS | Stigmatellin (35%) |
|  | Region 1917.1 | NODE1917.g10 | 28015 | 36076 | KS-AT-DH-MT-ER-KR-ACP | HR-PKS | Unknown |
|  | Region 1932.1 | NODE1932.g1 | 918 | 8711 | KS-AT-DH-MT-KR | PR-PKS | Decumbenonea/calbistrnA/calbistrin Cidecumbenone B/decumbenone cidioic acid moiely (15%) |
|  | Region 557.1 | NODE557.g14 | 47541 | 54958 | SAT-KS-AT-PT-ACP-ACP-ACP-Te | NR-PKS | Viniditoxin (22%) |
|  | Region 557.2 | NODE557.g16 | 58397 | 65418 | KS-AT-DH-ER-KR-ACP | NR-PKS | Unknown |
|  | Region 557.3 | NODE557.g19 | 73274 | 81143 | KS-AT-DH-MT-ER-KR | NR-PKS | Unknown |
|  | Region 1558.1 | NODE1558.g2 | 4896 | 11484 | KS-AT-PT-ACP-HTH-MT | NR-PKS | Higginsianin (80%) |
|  | Region 2091.1 | NODE2091.g1 | 582 | 7198 | SAT-KS-AT-PT-ACP-ACP-Te | NR-PKS | Unknown |
|  | Region 1046.1 | NODE1046 | 13257 | 33461 |  | Terpene | Terpenodole E (57%) |
|  | Region 1424.1 | NODE1424 | 17617 | 29486 |  | Terpene | Unknown |
|  | Region 1742.1 | NODE1742 | 1 | 10626 |  | Terpene | Unknown |
|  | Region 2029.1 | NODE2029 | 1 | 13942 |  | Terpene | Squalestatin S1 (40%) |
|  | Region 2139.1 | NODE2139 | 16371 | 37423 |  | Indole | Epoxy-Janthitreys (100%) |
|  | Region 1728.1 | NODE1728.g6 | 20846 | 24134 | AFD-P-Te | Other | Unknown |
| *O. camponoti-saundersi* | Region 11.1 | NODE11.g7 | 29205 | 43888 | A-C-A-P-C-P-C | NRPS | Unknown |
|  | Region 22.1 | NODE22.g13 | 61791 | 74360 | C-A-P-C-A-P-A-A-P-C | NRPS | Unknown |
|  | Region 955.1 | NODE955.g1 | 2346 | 13069 | A-P | NRPS | Unknown |
|  | Region 1034.4 | NODE1034.g140 | 575483 | 585691 | C-A-P | NRPS | Unknown |
|  | Tegion 1433.1 | NODE1433.g30 | 126574 | 129643 | A-P-Te | NRPS | Unknown |
|  | Region 1478.1 | NODE1478.g31 | 115402 | 118857 | A-Te | NRPS | Unknown |
|  | Region 333.1 | NODE333 | 176590 | 219430 |  | NRPS-like | Unknown |
|  | Region 934.2 | NODE934.g53 | 182243 | 184829 | A-P-Te | NRPS-like | Unknown |
|  | Region 1300.1 | NODE1300 | 18933 | 61521 |  | NRPS-like | Unknown |
|  | Region 1122.1 | NODE1122.g31 | 103590 | 111309 | A-C-MviM-WecE | NRPS,Other | Unknown |
|  | Region 1145.2 | NODE1145.g99 | 314443 | 319379 | CaiC-P-T | NRPS,Other | Dkxanthene (29%) |
|  | Region 1700.1 | NODE1700.g76 | 266250 | 269993 | A-P-Lys2b | NRPS,Other | Unknown |
|  | Region 1089.1 | NODE1089.g37 | 131785 | 143647 | KS-AT-DH-MT-KR-P-C-A-P-Te | PKS,NRPS | NG-391 (100%) |
|  | Region122.1 | NODE122.g14 | 77298 | 85441 | KS-AT-DH-MT-ER-KR-ACP | HR-PKS | Unknown |
|  | Region 776.1 | NODE776.g9 | 27172 | 34326 | KS-AT-DH-ER-KR-ACP | HR-PKS | Unknown |
|  | Region 876.1 | NODE867.g10 | 54948 | 62311 | KS-AT-DH-ER-KR-ACP | HR-PKS | Unknown |
|  | Region 1034.1 | NODE1034.g106 | 447875 | 456422 | KS-AT-DH-MT-ER-KR-ACP | HR-PKS | Chrodrimanin B (30%) |
|  | Region 1100.1 | NODE1100.g47 | 196534 | 204191 | KS-AT-DH-MT-ER-KR-ACP | HR-PKS | Unknown |
|  | Region 1145.3 | NODE1145.g100 | 319942 | 327962 | KS-AT-DH-MT-ER-KR-ACP | HR-PKS | Dkxanthene (29%) |
|  | Region 1233.1 | NODE1233.g25 | 155475 | 164023 | KS-AT-DH-ER-KR-ACP-Te | HR-PKS | Unknown |
|  | Region 1256.1 | NODE1256.g26 | 82973 | 90604 | KS-AT-ER-KR-ACP | HR-PKS | Pyranonigrin E (100%) |
|  | Region 1700.2 | NODE1700.g96 | 343391 | 350721 | KS-AT-DH-MT-ER-KR-ACP | HR-PKS | Unknown |
|  | Region 1700.3 | NODE1700.g99 | 359469 | 366618 | KS-AT-ER-KR-ACP | HR-PKS | Viriditoxin (22%) |
|  | Region 781.1 | NODE781.g12 | 59248 | 65622 | SAT-KS-AT-PT-Te | NR-PKS | Naphthopyrone (100%) |
|  | Region 834.1 | NODE834.g44 | 207459 | 213994 | SAT-KS-AT-PT-ACP-ACP-Te | NR-PKS | Unknown |
|  | Region 1034.2 | NODE1034.g109 | 460789 | 467341 | SAT-KS-AT-PT-ACP-HTH-MT | NR-PKS | Unknown |
|  | Region 1700.4 | NODE1700.g101 | 370973 | 378391 | SAT-KS-AT-PT-ACP-ACP-ACP-Te | NR-PKS | Unknown |
|  | Region 812.1 | NODE812 | 646730 | 688895 |  | PKS | Griseochelin (23%) |
|  | Region 1367.3 | NODE1367 | 443005 | 483961 |  | T3PKS | Unknown |
|  | Region 934.1 | NODE934 | 21926 | 42939 |  | Terpene | Unknown |
|  | Region 1034.3 | NODE1034 | 501033 | 522847 |  | Terpene | Clavaric acid (100%) |
|  | Region1145.1 | NODE1145 | 13970 | 55062 |  | Terpene | Terpendole E (100%) |
|  | Region 1367.1 | NODE1367 | 147021 | 165663 |  | Terpene | Unknown |
|  | Region 1367.2 | NODE1367 | 304155 | 324808 |  | Terpene | Unknown |
|  | Region 666.1 | NODE666 | 141649 | 162890 |  | Indole | Nodulisporic acid F (15%) |
|  | Region 1145.4 | NODE1145.g141 | 467879 | 472660 | P | Other | Unknown |
| *O. fusiformispora* | Region 4.1 | NODE4.g26 | 105241 | 109208 | A-P-Te | NRPS | Unknown |
|  | Region 11.1 | NODE11.g21 | 95619 | 105815 | C-A-P | NRPS | Unknown |
|  | Region 24.1 | NODE24.g2 | 11064 | 23552 | A-A-A-P-C-A-P-C | NRPS | Unknown |
|  | Region 39.1 | NODE39.g6 | 12451 | 16145 | A-MT-Te | NRPS | Unknown |
|  | Region 66.1 | NODE66.g8 | 27716 | 42127 | P-A-C-P-C-P-C | NRPS | Unknown |
|  | Region 130.1 | NODE130.g1 | 205 | 11020 | A-P | NRPS | Unknown |
|  | Region 305.1 | NODE305.g1 | 1324 | 4929 | A-Te | NRPS | Unknown |
|  | Region 267.1 | NODE267 | 1 | 30717 |  | NRPS-like | Unknown |
|  | Region 20.1 | NODE20.g12 | 46858 | 50440 | A-P-Lys2b | NRPS,Other | Unknown |
|  | Region 39.2 | NODE39.g7 | 17281 | 21095 | AspB-A-C | NRPS,Other | Unknown |
|  | Region 86.1 | NODE86.g7 | 30379 | 37631 | A-P-KS-AT-KR-ACP-Te | PKS,NRPS | Swainsonine (28%) |
|  | Region 2.1 | NODE2.g28 | 99809 | 119855 | KS-AT-DH-MT-KR-P-C-A-P-Te-MFS-Dehydrogenase-CYP-MT | PKS,NRPS,Other, | Lucilactaene (69%) |
|  | Region 5.1 | NODE5.g9 | 51700 | 59820 | KS-AT-DH-MT-ER-KR-ACP | HR-PKS | Ajudazol A (30%) |
|  | Region 37.2 | NODE37.g6 | 35326 | 43733 | KS-AT-DH-MT-ER-KR-ACP | HR-PKS | Unknown |
|  | Region 37.1 | NODE37.g4 | 23959 | 30498 | SAT-KS-AT-PT-ACP-HTH-MT | NR-PKS | Higginsianin B (80%) |
|  | Region 76.1 | NODE76.g13 | 43187 | 50658 | SAT-KS-AT-PT-ACP-ACP-ACP-Te | NR-PKS | YWA1 (100%) |
|  | Region 195.1 | NODE195.g5 | 24951 | 31504 | SAT-KS-AT-PT-ACP-ACP-Te | NR-PKS | Unknown |
|  | Region 280.1 | NODE280 | 1 | 29651 |  | PKS | Naphthopyrone (100%) |
|  | Region 46.1 | NODE46 | 1 | 34898 |  | T3PKS | Unknown |
|  | Region 105.1 | NODE105 | 19264 | 41402 |  | Terpene | Clavaric acid (100%) |
|  | Region 174.1 | NODE174 | 4439 | 25452 |  | Terpene | Squalestatin S1 (40%) |
|  | Region 377.1 | NODE377 | 1 | 16974 |  | Terpene | Unknown |
|  | Region 581.1 | NODE581 | 1 | 16974 |  | Terpene | Unknown |
|  | Region 947.1 | NODE947 | 1 | 10602 |  | Terpene | Unknown |
|  | Region 117.1 | NODE117 | 28671 | 49229 |  | Indole | Ergotamine (29%) |
|  | Region 136.1 | NODE130.g1 | 1 | 45712 |  | Fungal-RiPP-like | Unknown |
|  | Region 245.1 | NODE245 | 1 | 32175 |  | Fungal-RiPP-like | PneumocandinB0/A0 (6%) |
|  | Region 299.1 | NODE299 | 1 | 27993 |  | Fungal-RiPP-like | Unknown |
|  | Region 116.1 | NODE116.g7 | 30695 | 33984 | AFD-P-Te | Other | Unknown |
| *O．camponoti-floridani* | Region 1.5 | Scaffold1 | 4667491 | 4720594 |  | NRPS | Unknown |
|  | Region 2.1 | Scaffold2.g248 | 2271015 | 2284547 | C-A-P-A-P-A-P-C | NRPS | Unknown |
|  | Region 2.9 | Scaffold2 | 5074399 | 5124559 |  | NRPS | Unknown |
|  | Region 4.2 | Scaffold4.g132 | 553878 | 557360 | A-Te | NRPS | Unknown |
|  | Region 5.3 | Scaffold5.g372 | 2629984 | 2633464 | A-Te | NRPS | Unknown |
|  | Region 5.4 | Scaffold5.g465 | 3097710 | 3107842 | A-P | NRPS | Unknown |
|  | Region 6.1 | Scaffold6.g1 | 2927 | 6778 | C-A-Te | NRPS | Unknown |
|  | Region 6.3 | Scaffold6 | 2183032 | 2226313 |  | NRPS-like | Unknown |
|  | Region 4.1 | Scaffold4.g35 | 125957 | 131003 | CaiC-P-T | NRPS,Other | Unknown |
|  | Region 1.6 | Scaffold1 | 4929987 | 4981096 |  | PKS,NRPS | NG-391 (66%) |
|  | Region 1.1 | Scaffold1.g22 | 71971 | 83056 | KS-AT-DH-MT-ER-KR-ACP | HR-PKS | Ajudazol A (30%) |
|  | Region 2.5 | Scaffold2.g550 | 3533425 | 3541316 | KS-AT-DH-ER-KR-ACP | HR-PKS | Unknown |
|  | Region 2.7 | Scaffold2.g727 | 427780 | 4285273 | KS-AT-DH-MT-ER-KR-ACP | HR-PKS | Unknown |
|  | Region 3.2 | Scaffold3.g21 | 108268 | 115320 | KS-AT-ER-KR-ACP | HR-PKS | Unknown |
|  | Region 5.2 | Scaffold5.g175 | 1837675 | 1845037 | KS-AT-DH-ER-KR-ACP | HR-PKS | Unknown |
|  | Region 3.3 | Scaffold3.g25 | 160810 | 167953 | KS-AT-MT-ER | PR-PKS | Unknown |
|  | Region 2.3 | Scaffold2.g376 | 2795667 | 2802028 | SAT-KS-AT-PT-ACP-HTH-MT | NR-PKS | Higginsianin B (80%) |
|  | Region 3.1 | Scaffold3.g19 | 97233 | 104695 | SAT-KS-AT-PT-ACP-ACP-ACP-Te | NR-PKS | 6-hydroxymellein (33%) |
|  | Region 7.1 | Scaffold7.g388 | 2310819 | 2317328 | SAT-KS-AT-PT-ACP-ACP-Te | NR-PKS | Neosartorin (10%) |
|  | Region 1.3 | Scaffold1 | 2583651 | 2624736 |  | T3PKS | Unknown |
|  | Region 1.2 | Scaffold1 | 1655198 | 1672863 |  | Terpene | Unknown |
|  | Region 1.4 | Scaffold1 | 2996681 | 3017826 |  | Terpene | Unknown |
|  | Region 2.2 | Scaffold2 | 2446850 | 24664507 |  | Terpene | Unknown |
|  | Region 2.4 | Scaffold2 | 3482551 | 3502656 |  | Terpene | Squalestatin S1 (40%) |
|  | Region 2.8 | Scaffold2 | 4373330 | 4394199 |  | Terpene | Unknown |
|  | Region 5.1 | Scaffold5 | 1631619 | 1642714 |  | Terpene | Terpendole E (57%) |
|  | Region 5.5 | Scaffold5 | 3184008 | 3205825 |  | Terpene | Unknown |
|  | Region 6.2 | Scaffold6 | 155936 | 176571 |  | Indole | Unknown |
|  | Region 2.6 | Scaffold2.g714 | 4216326 | 4221913 | P | Other | Unknown |

Table S6. Overview of biosynthetic gene clusters in the genomes of the twelve studied fungi.

| **Species (NCBI)** | **No of cluster** | **NRPS** | **NPRS-like** | **PKS (Total)** | **HR-PKS** | **PR-PKS** | **NR-PKS** | **T3PKS** | **Terpene** | **Hybrid PKS-NRPS** | **Hybrid NRPS-Other** | **Hybrid PKS-NRPS-Other** | **Hybrid PKS-Other** | **Other** |
| --- | --- | --- | --- | --- | --- | --- | --- | --- | --- | --- | --- | --- | --- | --- |
| *O. contiispora* | 27 | 6 |  | 10 | 3 | 3 | 4 | 1 | 6 | 2 | 1 |  |  | 1 |
| *O. subtiliphialida* | 25 | 8 |  | 7 | 4 |  | 3 | 1 | 5 | 1 | 2 |  |  | 1 |
| *O. camponoti-rufipedis* | 26 | 6 |  | 5 | 2 |  | 3 | 1 | 7 | 1 | 6 |  |  |  |
| *O. camponoti-floridani* | 29 | 7 | 1 | 9 | 5 | 1 | 3 | 1 | 7 | 1 | 1 |  |  | 2 |
| *O. satoi* | 28 | 7 |  | 9 | 5 |  | 4 | 1 | 3 | 1 | 4 | 1 |  | 2 |
| *O. polyrhachis-furcata* | 28 | 8 |  | 13 | 7 |  | 6 |  | 3 | 1 | 1 |  |  | 2 |
| *O. flabellata* | 32 | 8 |  | 14 | 9 | 1 | 4 | 1 | 3 | 2 | 2 |  |  | 2 |
| *O. camponoti-saundersi* | 36 | 6 | 3 | 15 | 10 |  | 4 | 1 | 5 | 1 | 3 |  |  | 2 |
| *O. acroasca* | 25 | 7 |  | 10 | 5 |  | 5 |  | 4 | 1 | 3 |  |  |  |
| *O. camponoti-leonardi* | 36 | 8 |  | 14 | 9 |  | 5 | 1 | 8 | 1 | 2 |  | 1 | 1 |
| *O. fusiformispora* | 29 | 7 | 1 | 6 | 2 |  | 3 | 1 | 5 | 1 | 2 | 1 |  | 5 |
| *O. unilateralis* | 26 | 6 | 2 | 9 | 3 | 1 | 5 |  | 4 | 1 | 2 |  |  | 2 |


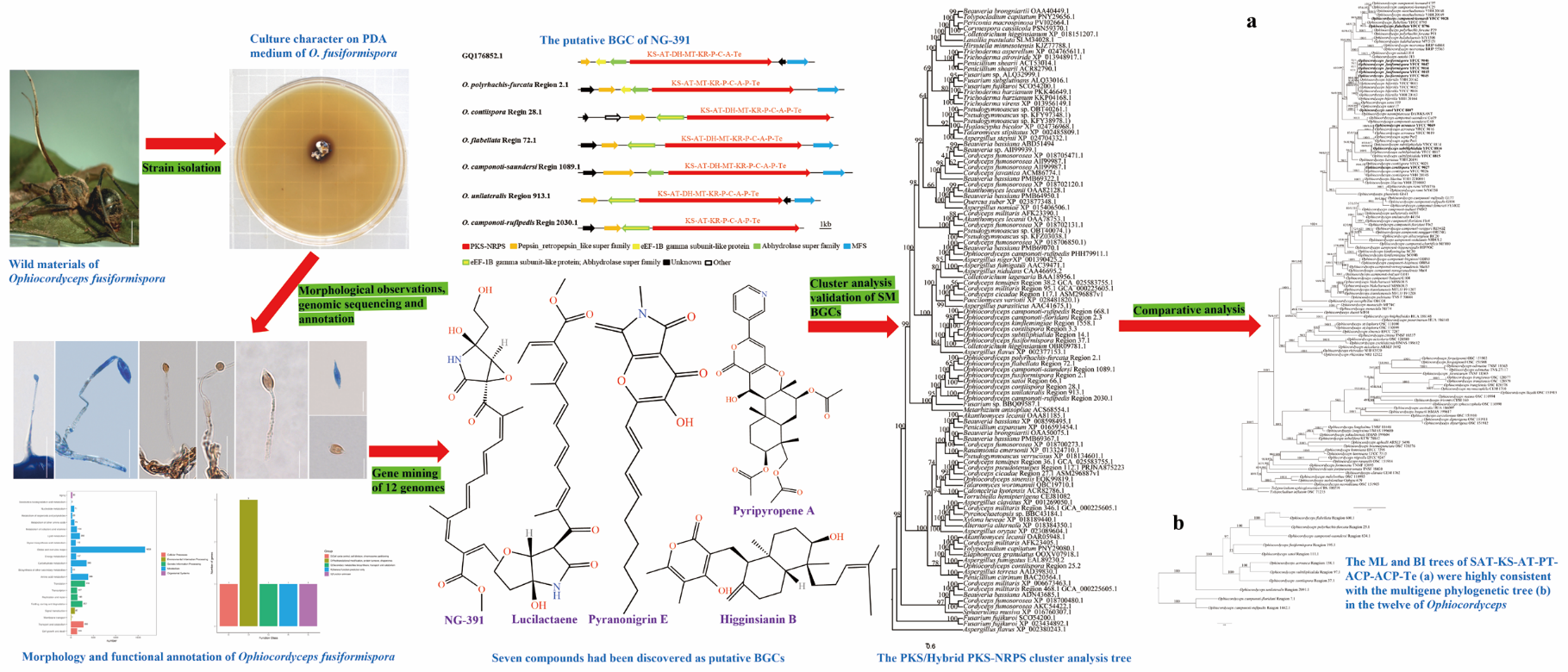


Figure S1. Technology roadmap.


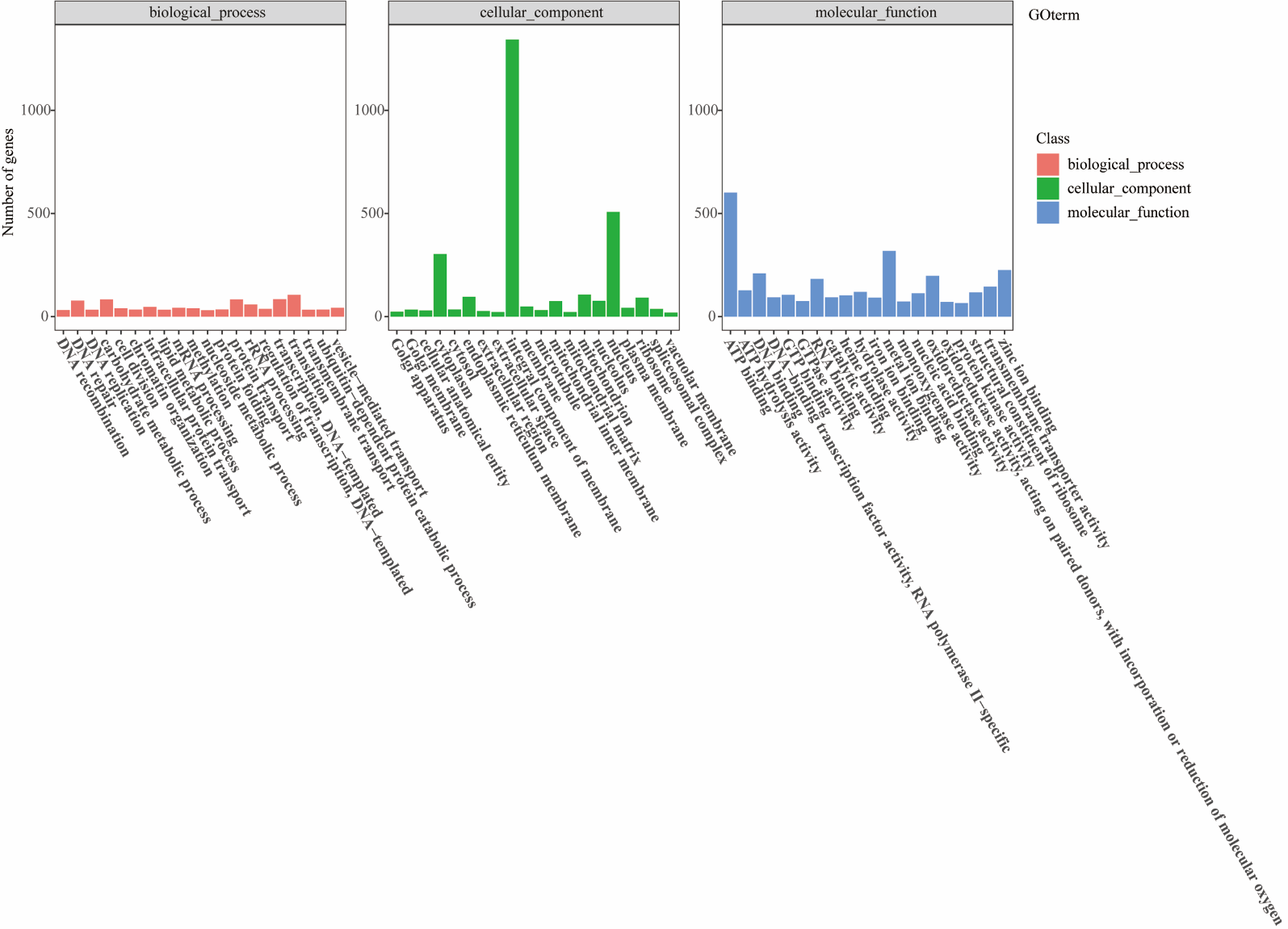


Figure S2. The biological function of functional genes involved in the annotation of *O. satoi* in the GO analysis.


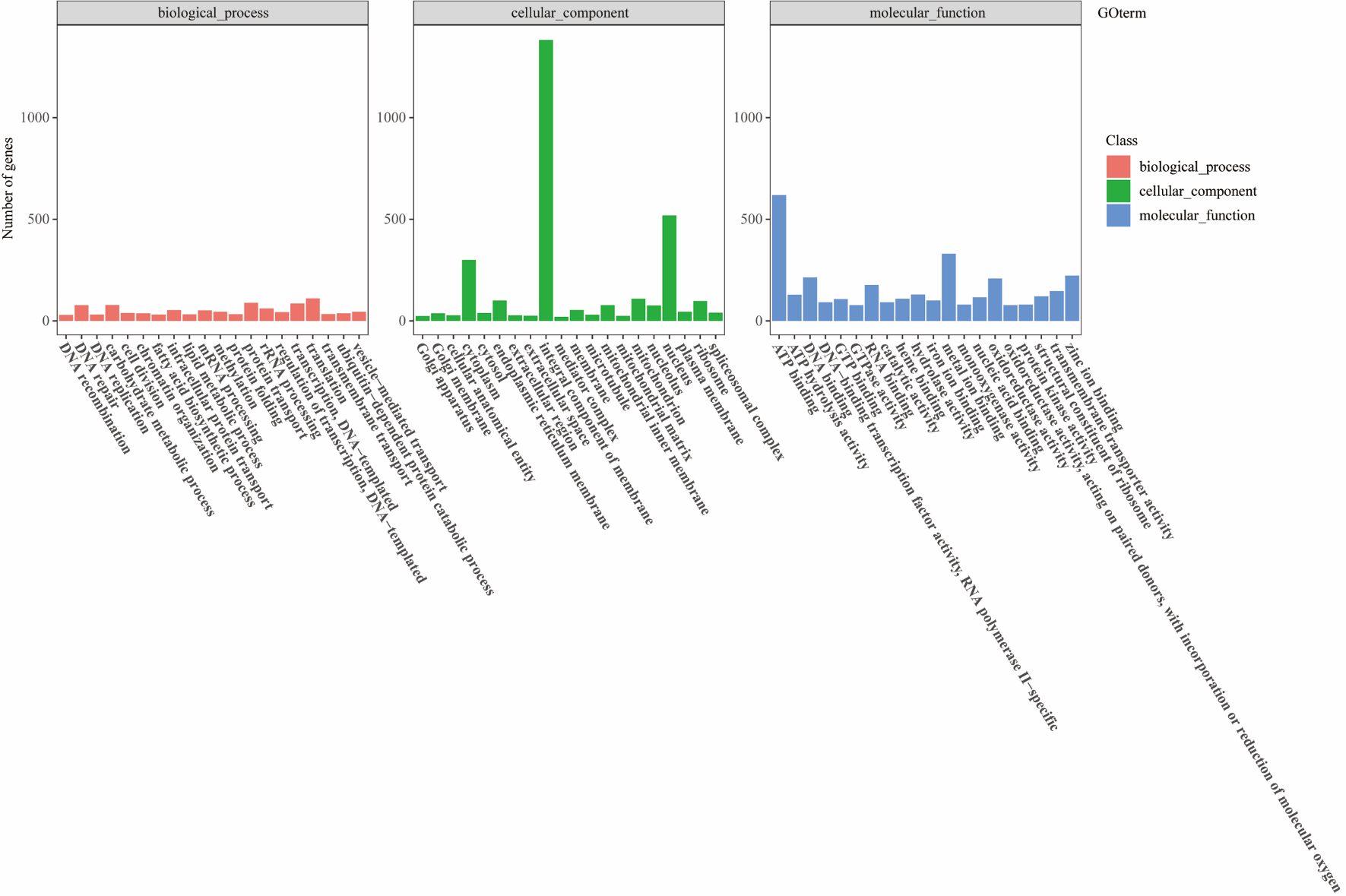


Figure S3. The biological function of functional genes involved in the annotation of *O. flabellata* in the GO analysis.


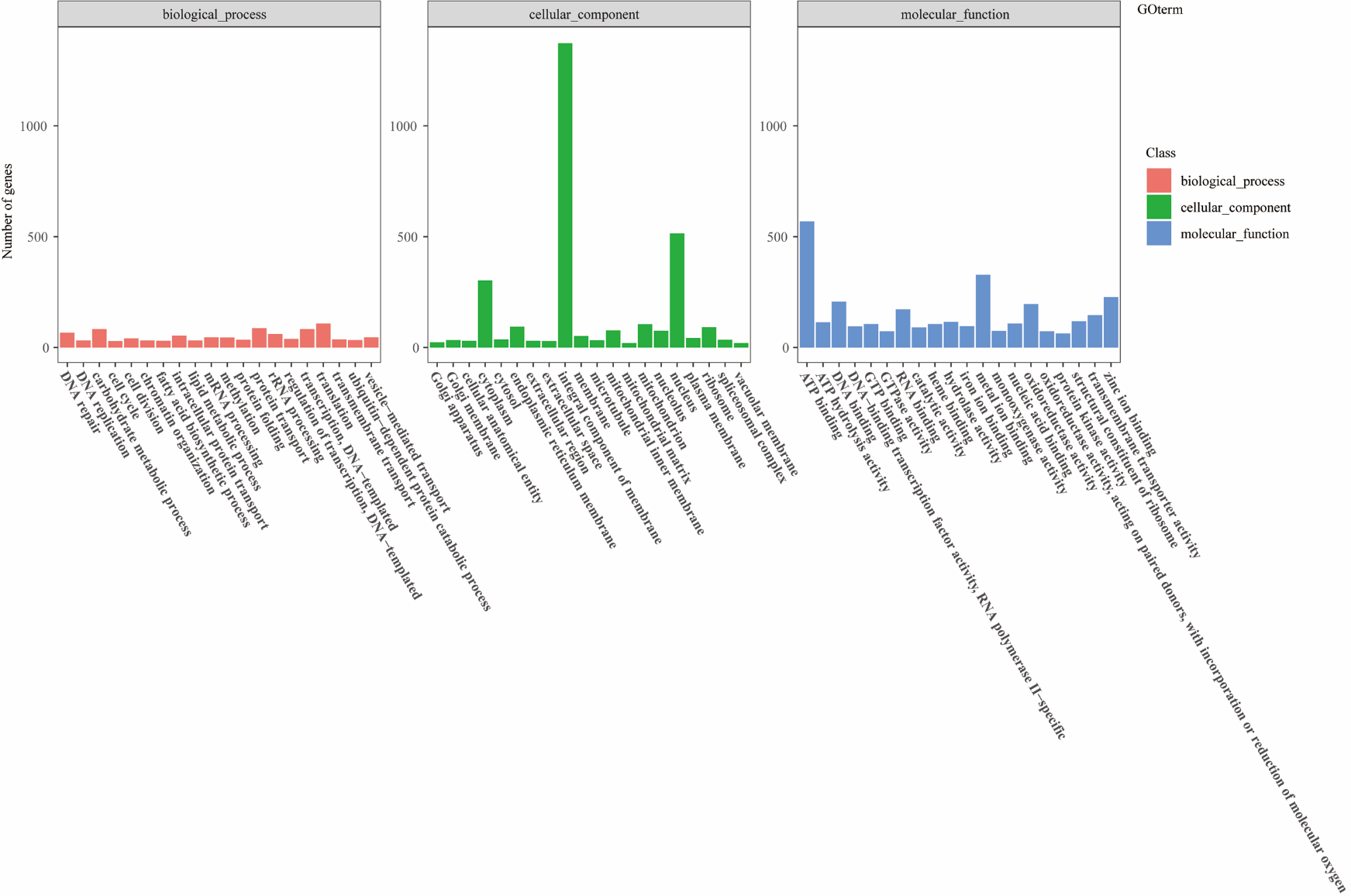


Figure S4. The biological function of functional genes involved in the annotation of *O. acroasca* in the GO analysis.


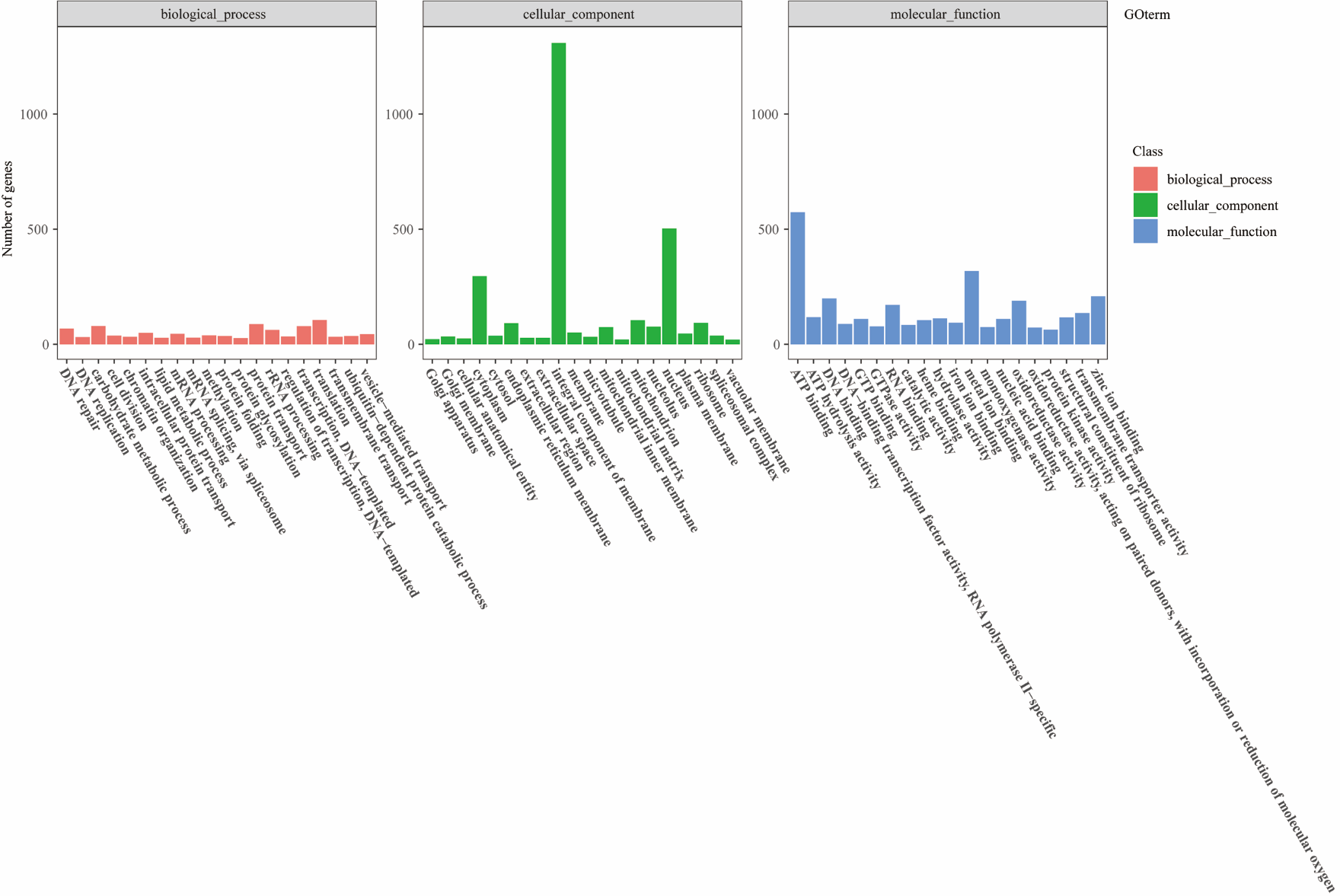


Figure S5. The biological function of functional genes involved in the annotation of *O. subtiliphialida* in the GO analysis.


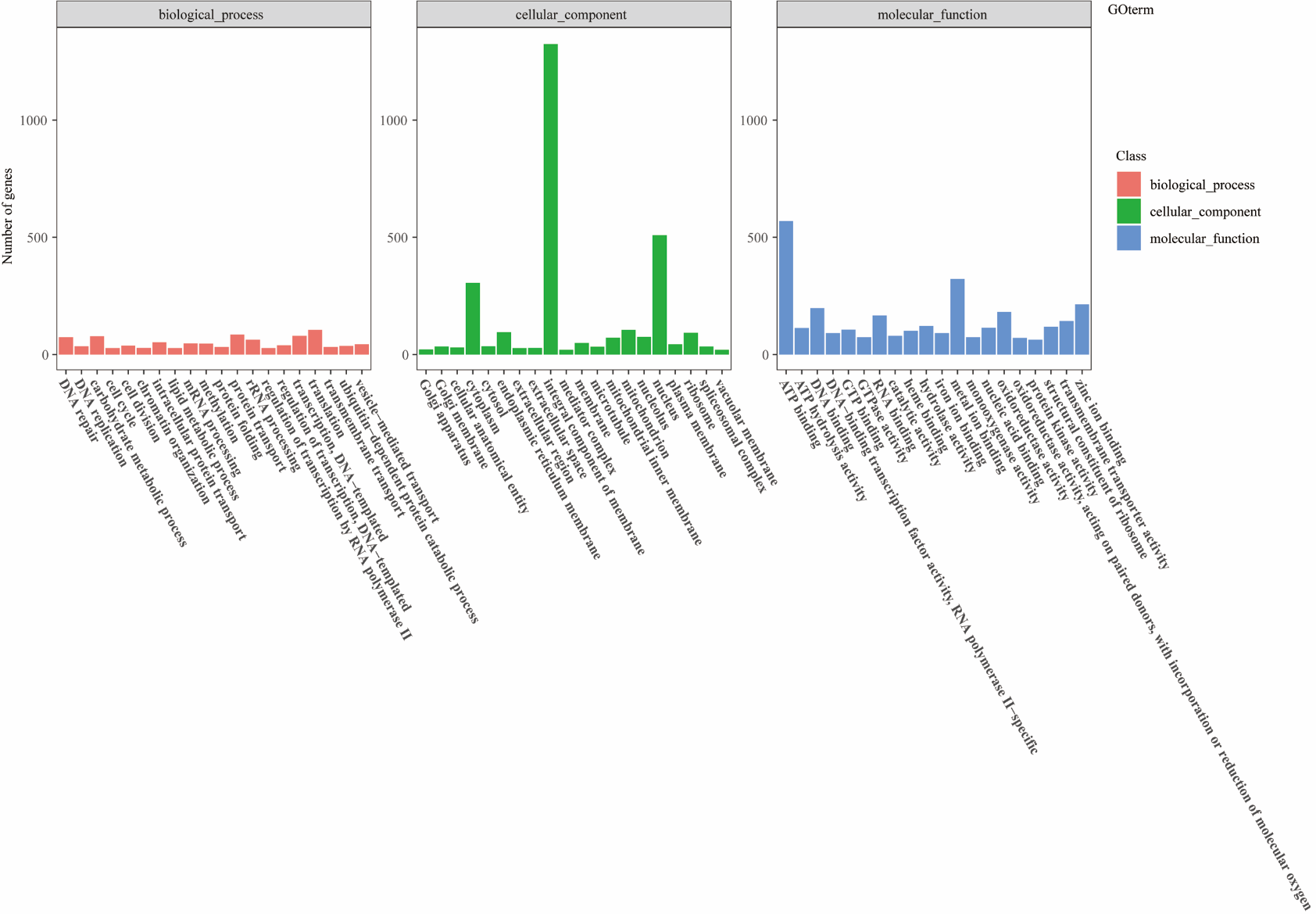


Figure S6. The biological function of functional genes involved in the annotation of *O. contiispora* in the GO analysis.


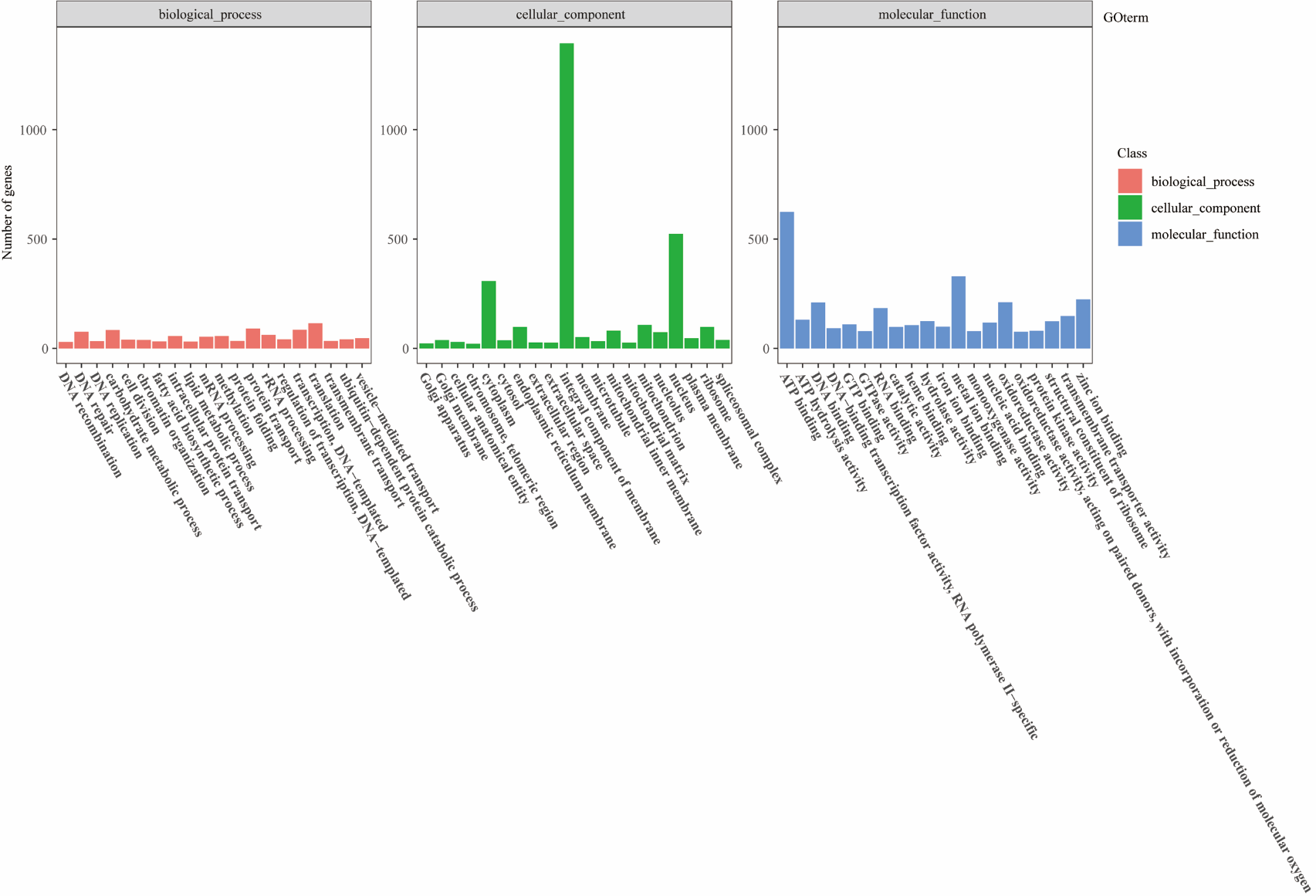


Figure S7. The biological function of functional genes involved in the annotation of *O. camponoti-leonardi* in the GO analysis.


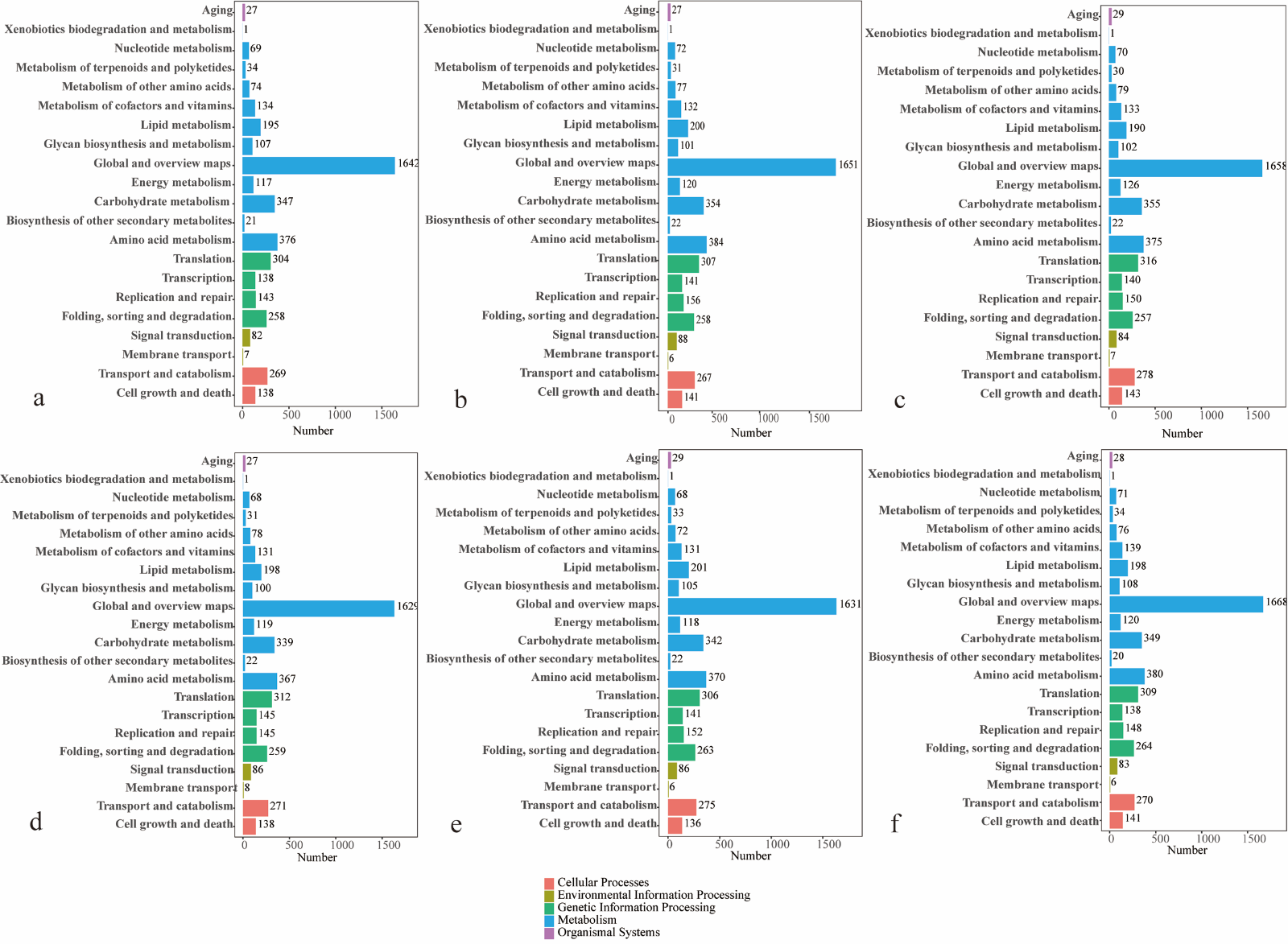


Figure S8. The KEGG analysis of seven species of *O. unilateralis* sensu lato. Values represent the number of genes. a: *O. satoi*; b: *O.* *flabellate*; c: *O. acroasca*; d: *O.* *subtiliphialida*; e: *O. contiispora*; f: *O. camponoti-leonardi*.


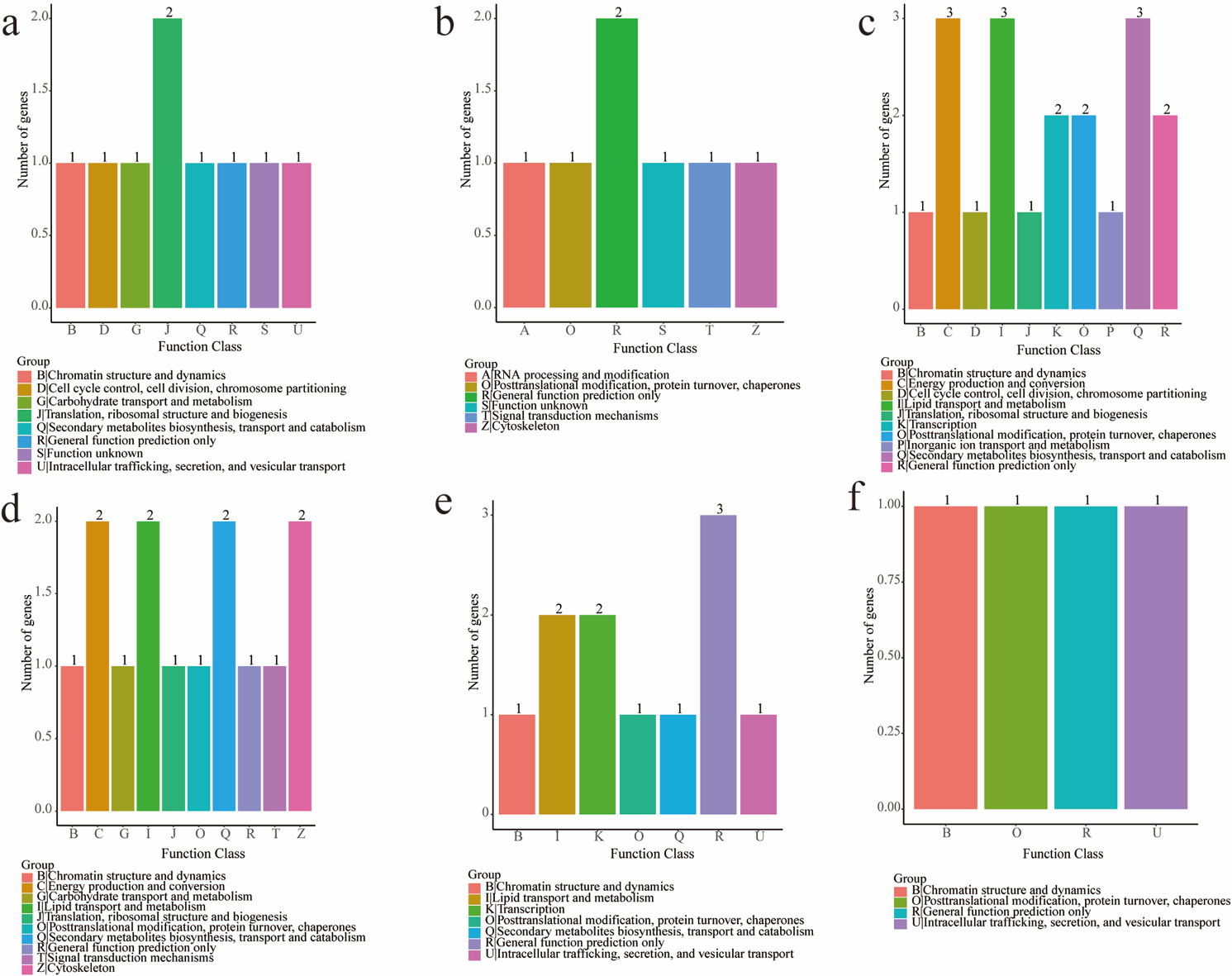


Figure S9. The KEGG analysis of seven species of *O. unilateralis* sensu lato. Values represent the number of genes. a: *O. satoi*; b: *O.* *flabellate*; c: *O. acroasca*; d: *O.* *subtiliphialida*; e: *O. contiispora*; f: *O. camponoti-leonardi*.
